# 20S and 26S proteasome-binding proteins of the rabbit brain: A proteomic dataset

**DOI:** 10.1016/j.dib.2021.107276

**Published:** 2021-08-11

**Authors:** Olga Buneeva, Arthur Kopylov, Svetlana Kaloshina, Victor Zgoda, Alexei Medvedev

**Affiliations:** Institute of Biomedical Chemistry, 10 Pogodinskaya street, Moscow 119121 Russian Federation

**Keywords:** Proteasome isolation, Rabbit brain, Proteomic profiling, Proteasome core and regulatory particles, Proteasome-binding proteins, Multifunctional proteins

## Abstract

Fractions of 26S and 20S proteasomes isolated from the rabbit brain by the method of salt fractionation (salt-induced precipitation) contain intrinsic proteasome proteins responsible for assembly of the core particle and regulatory particle of proteasome and also proteasome-binding proteins. These proteasome-binding proteins include components of the ubiquitin-proteasome system, some ubiquitinated proteins, as well as cytoskeleton components, protective proteins, regulators of gene expression, cell division, and differentiation, and multifunctional proteins (mainly, glycolytic enzymes: glyceraldehyde-3-phosphate dehydrogenase (GAPDH), aldolase, pyruvate kinase, etc.). The multifunctional proteins also known as “moonlighting proteins” are involved in various (regulatory) processes in the cell and obviously represent important components of the proteasome interactome rather than contaminants of the 26S and 20S proteasome fractions.

## Specifications Table

**Table:** 

Subject	Biology
Specific subject area	Proteasome isolation, proteomic analysis, and protein identification
Type of data	Tables Figures
How data were acquired	Raw data were acquired using a high-resolution hybrid mass spectrometer Orbitrap Fusion in a positive ionization mode and coupled with an Ultimate RSLC 3000 nano-flow liquid chromatography system.
Data format	Analyzed LC-MS raw data file were converted in a MGF peak list format using MSConvert (Proteowizard) and passed to search engine X!Tandem Vengeance (version 2015.12.15.2) for identification.
Parameters for data collection	Data were obtained at a resolution of 60 K in MS1 level and 15 K at MS2 and MS3 levels. Precursor and primary fragment ions were decomposed in an HCD mode and isolated using quadrupole. Asymmetric tolerance of −3/+7 ppm was applied for fragment ions before triggering the MS3 fragmentation.
Description of data collection	Rabbit brain26S and 20S proteasomes were isolated by differential centrifugation of the brain homogenate followed by ammonium sulfate fractionation. Proteins of the 26S and 20S proteasome fractions were extracted with a mixture of chloroform–methanol, trypsinized and then high resolution mass spectrometry analysis was performed.
Data source location	Institute of Biomedical Chemistry, Moscow, Russia
Data accessibility	Data files exported after identification of proteins and peptides are available at https://data.mendeley.com/datasets/g9tpsdctt4/1. Tables with processed results are available as Supplementary Materials to this paper.

## Value of the Data

• Rabbit brain is a valuable source of proteasomes to study not only important features of proteasomal degradation of brain proteins but also interaction of proteasomal core particle proteins with non-proteasomal proteins involved in formation of a proteasome interactome.
• The fractions of 20S and 26S proteasomes isolated from the rabbit brain by salt-induced precipitation followed by intensive washing contain both intrinsic proteasomal proteins and proteins associated with these particles, which form the proteasome interactome. The abundance of some multifunctional proteins associated with proteasomes is comparable with the abundance of proteasomal core proteins.
• The data on 20S and 26S proteasome-binding proteins would be valuable for further studies of both degradation of particular (ubiquitinated and non-ubiquitinated) proteins in proteasomes and a role of associated proteins in the proteasome functioning in health and disease.

## 1 Data Description

Four independent proteomic experiments using 26S and 20S proteasomal fractions isolated from four rabbit brains were performed. Proteins extracted from these fractions were analyzed by LC-MS/MS. Raw mass spectrometry data were deposited in the Mendeley Data Repository. Supplementary materials in the Mendeley Data Repository are presented as data files exported after RAW data processing using the proteomic search engine. Data Folder “Initial Data per Animal” contains 12 files of the main post-search data distributed in three sheets: (1) Peptide Identification Summary; (2) Protein Identification Summary; and (3) Peptide Spectrum Matching Summary. Data sheets contain information about the confidence score, theoretical and sequenced peptides; possible protein inference and detected modifications in amino acid sequences. The “Protein Identification Summary” data sheet covers the information regarding the calculated spectrum counting scores defined in terms of intensity, emPAI and NSAF coefficient (including both absolute arbitrary and relative estimations). The “Peptide Spectrum Matching Summary” data sheet comprehends information of PSMs relevant to identified peptides (including modified peptides) with the defined measurement errors (in ppm and Da) and the detected charge state, as well as the indexed position of peptide within protein amino acid sequence and the annotated by MS peptide sequence. The folder “Combined Data with Proteins Distribution” contains a file, which is a compilation of the proteome profiles of animals under the study, and contains the essential information about proteins distribution, frequencies and semi-quantitative (based on the NSAF coefficient) of proteins over the study population. The proteins identified at least in three proteomic experiments are listed in Supplementary Table 1 (the 26S proteasome fraction) and 2 (the 20S proteasome fraction). The proteins identified in two proteomic experiments are listed in Supplementary Tables 3 and 4 (the 26S and 20S proteasome fractions, respectively).

**Fig. 1:**
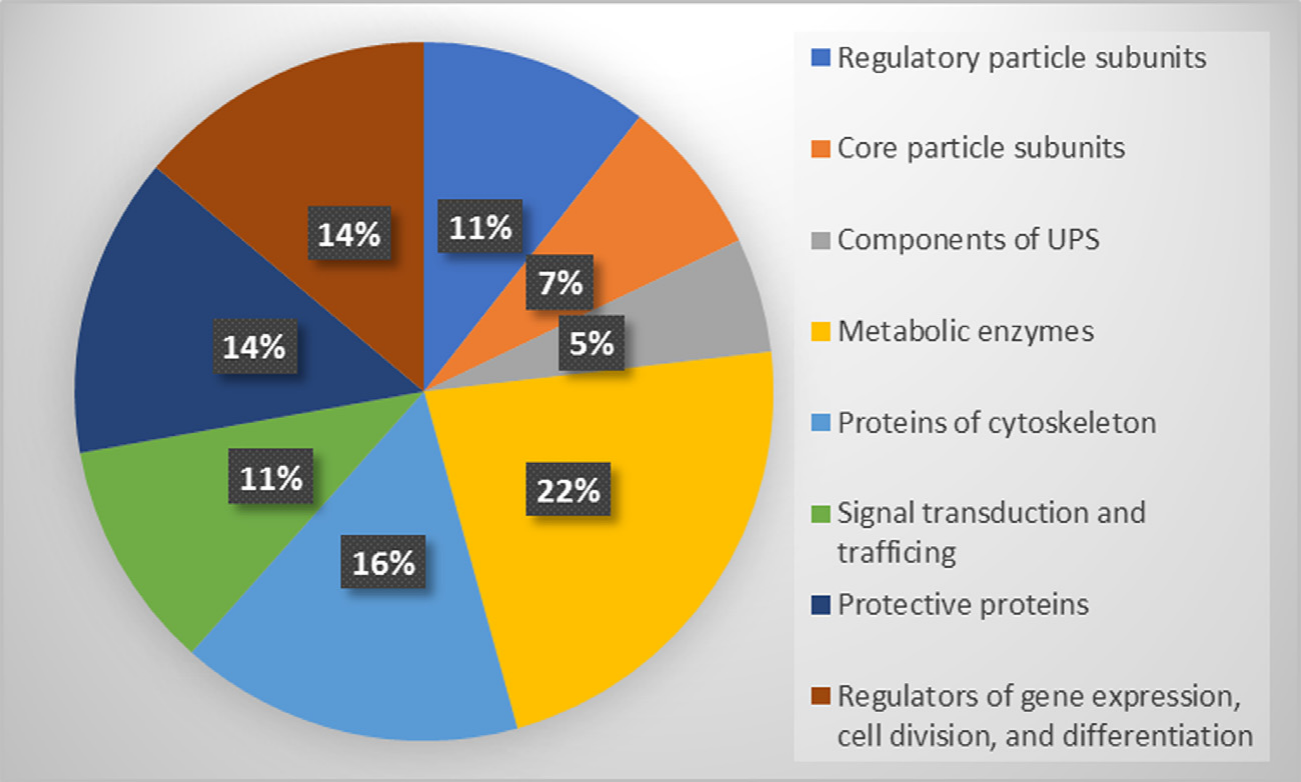
Proteomic profile of the 26S proteasome fraction (rabbit brain).

**Fig. 2:**
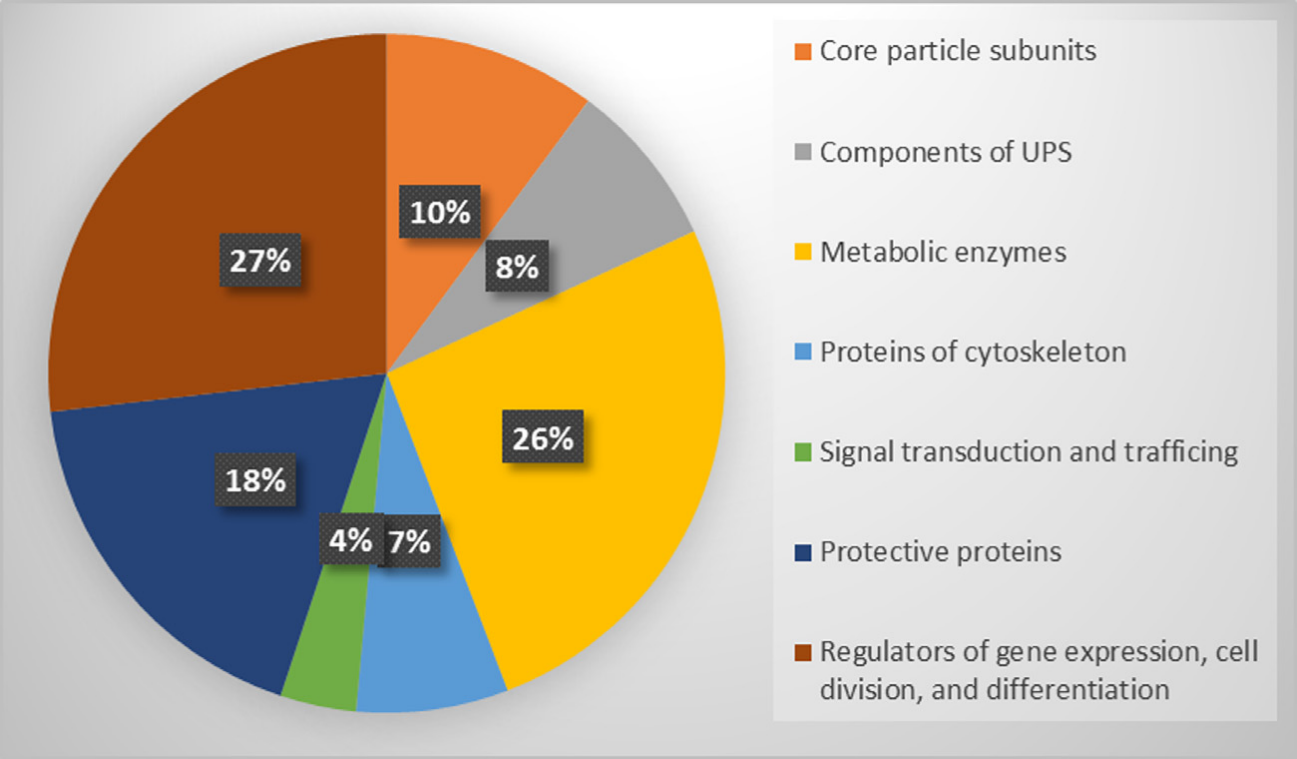
Proteomic profile of 20S proteasome fraction (rabbit brain).

**Fig. 3:**
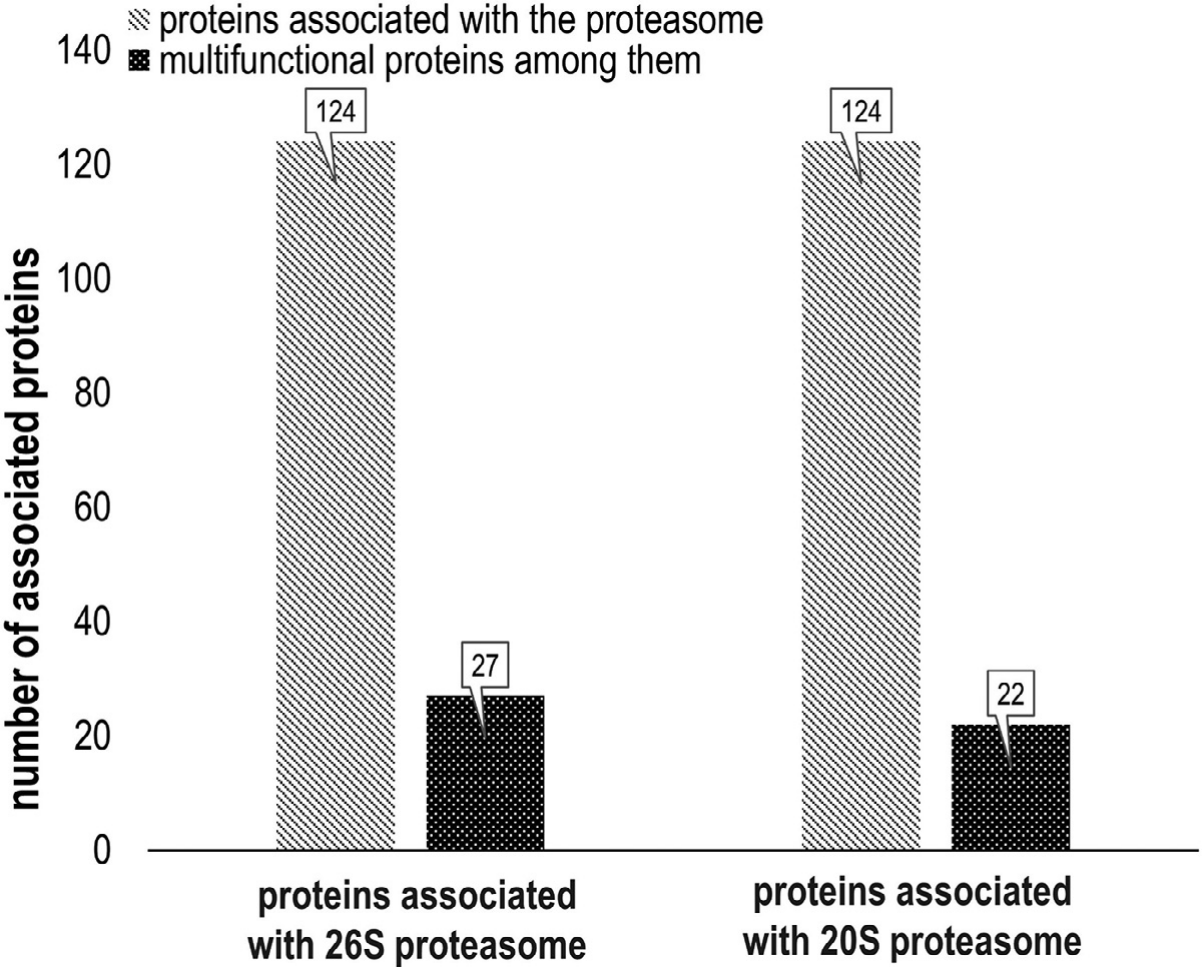
Ratio of multifunctional proteins in 26S and 20S proteasome fractions from rabbit brain: greay columns indicate the number of proteins associated with proteasomose; black columns indicate the fraction of multifunctional proteins among them.

**Fig. 4:**
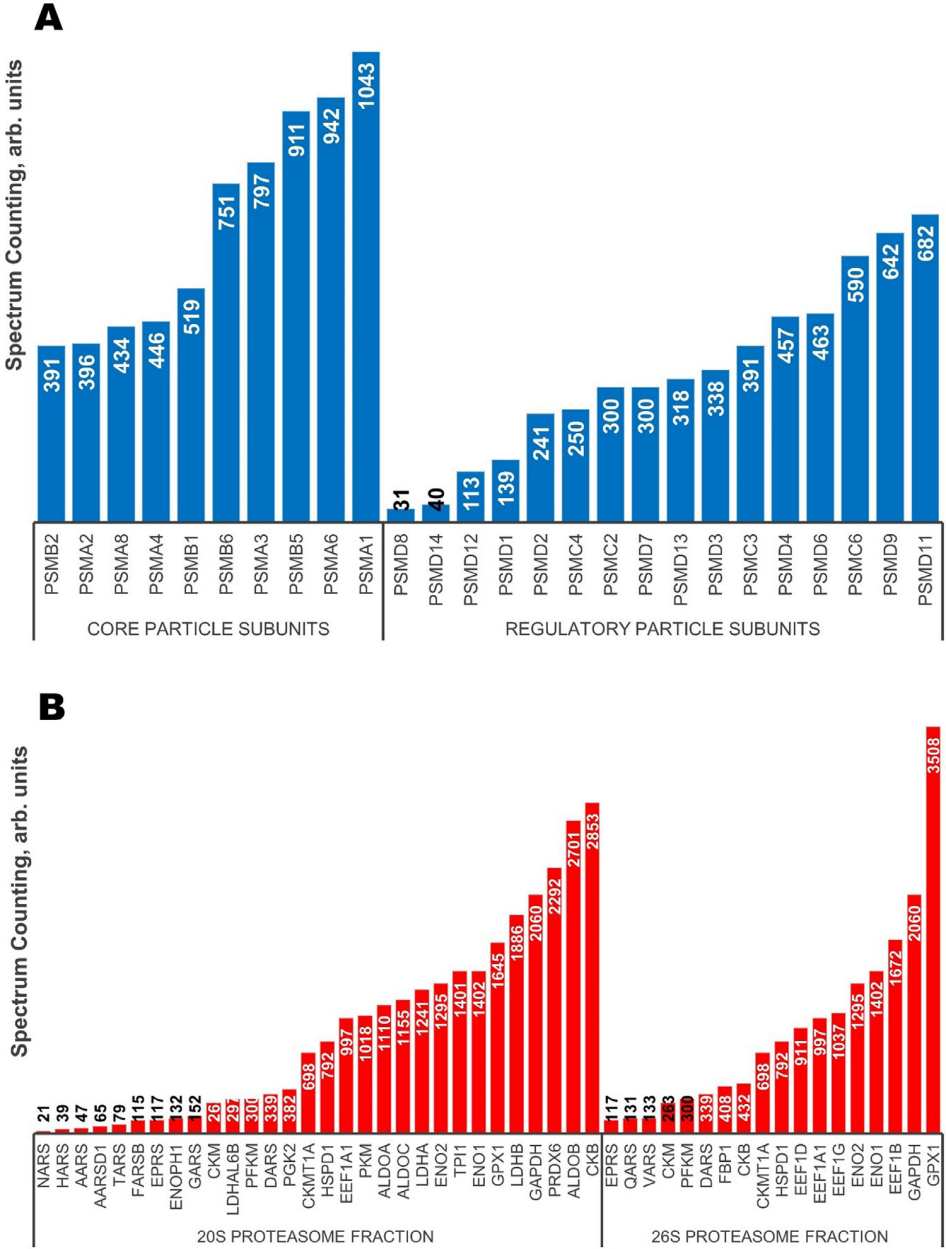
Comparative spectrum counting of proteasomal core particle proteins (A) and multifunctional proteins (B) identified in the fractions of rabbit brain 26S proteasomes and 20S proteasomes.

Proteomic profiling of the 26S and 20S proteasome fractions isolated from rabbit brain by the method of salt-induced precipitation revealed 151 and 138 proteins, respectively (Supplementary Tables 1 and 2). In the case of the 26S proteasome fraction, regulatory particle subunits accounted for 11%, and core particle subunits – for 7% of the total number of proteins. The 20S proteasome fraction was free of the components of regulatory subunit, and the part of core particle subunits was equal to 10%. Both fractions contained other components of ubiquitin-proteasome system, metabolic enzymes, components of cytoskeleton, protective proteins, proteins of signal transduction and trafficking, and regulators of gene expression, cell division and differentiation (see Figs. 1 and 2). A high proportion of proteins associated with both proteasome fractions (Fig. 3) (mainly, glycolytic enzymes: glyceraldehyde-3-phosphate dehydrogenase (GAPDH), aldolase, pyruvate kinase, etc.) was represented by so called moonlighting proteins (Supplementary Tables 1 and 2, highlighted in bold) [1], [2], [3], [4], [5]. These multifunctional proteins are known by their intrinsically unfolded domains [6,7], which could be a prerequisite for their proteolysis directly by the core proteasome particle without ubiquitination [8,9]. In this context, abundance of some of the multifunctional proteins is comparable to the abundance of the proteasomal particle core proteins (Fig. 4).

## 2 Experimental Design, Materials and Methods

### 2.1 Isolation of rabbit brain 26S and 20S proteasomes

Four adult male albino New Zealand White rabbits, with body mass of 3.5–5.0 kg, were decapitated after intramuscular injection of zoletil (10 mg/kg). Fractions of 26S and 20S proteasomes were isolated from rabbit brain according to the method by Sharova et al. [10] with minor modifications. The extracted brain tissue was homogenized in Buffer А (30 mМ Tris–HCl, pH 7.5, 100 mМ NaCl, 1 mМ EDТА, 1 mМ dithiothreitol, 10% glycerol (v/v), 5 mМ MgCl_2_, 2 mМ АТP, 10 mМ Na_2_S_2_O_5_, a protease inhibitor cocktail (1 ml/l, Merck) under cooling (SilentCrusher S (Heidolph), 70,000 rpm; tissue: buffer ratio 1;3, w/v). The homogenate was centrifuged (90 min, 105,000 *g*, 4 °C). After addition of phosphocreatine and creatine kinase (final concentrations of 10 mM and 10 µg/ml respectively) the resultant supernatant was incubated 30 min at 37 °С. The mixture was fractionated with ammonium sulfate in three stages. Within 30 min ammonium sulfate was added by portions upon stirring at 4 °С up to 38% saturation, kept stirred for 30 min at 4 °С and centrifuged (10,000 *g*, 30 min, 4 °C) to separate the pellet, containing a pool of 26S proteasomes. This pellet was resuspended in Buffer В (20 mМ Tris–HCl, pH 7.5, 1 mМ EDТА, 1 mМ dithiothreitol, 50% glycerol (v/v), 5 mМ MgCl_2_, 2 mМ АТP, 10 mМ Na_2_S_2_O_5_, a protease inhibitor cocktail (1 ml/l). After pellet resuspension and addition of phosphocreatine and creatine kinase (final concentrations of 10 mM and 10 µg /ml, respectively) the mixture was incubated 30 min at 37 °С. The resultant fraction of 26S proteasomes (protein content of approximately 2 mg/ml) was also stored at −20 ^°^С.

The supernatant from the previous step (38% saturated ammonium sulfate supernatant) was used for isolation of 20S proteasomes. Ammonium sulfate was gradually added to supernatant up to 42% saturation, kept stirred for 30 min and centrifuged under the same conditions to separate the pellet containing contaminant proteins. The resultant supernatant was used to obtain the 20S proteasome fraction which was isolated by gradual adding ammonium sulfate up to 70% saturation followed by centrifugation (10,000 *g*, 30 min, 4 °C). The final pellet was resuspended in Buffer C (20 mМ Tris–HCl, pH 7.5, 1 mМ EDТА, 1 mМ dithiothreitol, 50% glycerol (v/v), 10 mМ Na_2_S_2_O_5_, a protease inhibitor cocktail (1 ml/l). The 20S proteasome fraction (protein content of approximately 2 mg/ml) was also stored at −20 °С.

### 2.2 Sample preparation for proteomic analysis

Proteins were extracted with a mixture of chloroform – methanol [11]. Initially, methanol was added to the protein sample (1:2.5), and the sample was vortexed vigorously for 30 s, then chloroform was added (the volume equal to the initial volume of the protein sample); the resultant mixture was vortexed again and centrifuged at 23,755 *g* at 20 °C for 2 min. The upper layer was discarded and a 3-fold excess (versus initial sample) of methanol was added, then the sample was vortexed and centrifuged at 20 °C for 3 min. The supernatant was aspirated, and the resultant sediment was dried under air for 20–30 min, modified, and digested on filters as described in [12]. Briefly, the sediment of precipitated proteins was dissolved in 200 µl of lysis buffer (0.1 M Tris–HCl, pH 8.5, 5 M urea, 0.015 M dithiothreitol). The samples were pipetted onto the Vivaspin 500 ultrafiltration devices (10 kDa) (GE Healthcare, USA) and centrifuged at 1485 *g* at 20 °C for 40 min. Then 100 µl of alkylation solution (0.05 M iodoacetamide in washing solution (0.05 M ammonium bicarbonate, pH 8.0)) was added and the filters were incubated at 20 °C for 30 min in darkness. The filters were centrifuged under the same conditions for 40 min and washed twice with 200 µl of washing solution (each time centrifuged under the same conditions for 40 min). After that trypsin of sequencing grade was added at a ratio of 1:100 = total mass of enzyme: total mass of protein (as the amount of total protein was approximately 200 µg, 2 µg of trypsin was used) and the filters were incubated overnight at 37 °C using a Thermostat 5320 (Eppendorf, Germany). After centrifugation under the same conditions for 40 min the samples were washed with 100 µl of 0.5 M NaCl.

### 2.3 Liquid chromatography and mass spectrometry

Liquid chromatography of tryptic peptides was performed using the Ultimate 3000 RSLCnano system. Peptides in a volume of 2 µL (totally 1 µg of peptides) were loaded on an enrichment column Acclaim µ-Precolumn (0.5 mm × 3 mm, 5 µm) (Thermo Scientific, USA) in a mobile phase comprised of 0.1% formic acid and 0.03% acetic acid at a flow rate 15 µL/min. After 3 min of loading, peptides were departure from the enrichment column and separated on the Acclaim Pepmap® C18 column (75 µm × 100 mm, 2 µm) (Thermo Scientific) at a flow rate 0.3 µL/min in a linear gradient of the mobile phase A (water with 0.1% formic acid and 0.03% acetic acid) and the mobile phase B (acetonitrile with 0.1% formic acid and 0.03% acetic acid). The eluting scheme started from 2% to 37% of the mobile phase B during 45 min, then column washing in 90% of mobile phase B for the next 8 min, followed by the column equilibration under the starting condition for the next 15 min.

Detection of peptides was performed using an ultra-high resolution Orbitrap Fusion (Thermo Scientific) mass spectrometer as described previously [11] in a positive ionization mode. The instrument was equipped with the NSI ionization source with the capillary voltage adjusted to 2.2 kV and drying gas temperature adjusted to 280 °C. Precursor ions were scanned in a range from 400 *m/z* to 1200 *m/z* with a maximum integration time of 80 ms. Ions filtered by the charge state from *z* = 2+ to *z* = 6+ were isolated by a quadrupole within ±1.5 *m/z* window and taken for dissociation using the HCD mode (high-energy collision-induced dissociation) at 27% of normalized energy. The ubiquitin mass tags of ΔM = 114.0429 *u* (corresponding to GG) and ΔM = 383.2281 *u* (corresponding to LRGG) were detected using the MS3 synchronous fragment ions selection mode with an asymmetric mass tolerance.

### 2.4 Protein identification and go annotation

The MSConvert (Proteowizard) tool was utilized to convert RAW files into MGF files suitable for proteins identification using X!Tandem Vengeance (version 2015.12.15.2) search engine embedded in the SearchGUI version 3.3.0 interface [13,14]. Identification was conducted against target proteins database specified to *Oryctolagus cuniculus* (16,915 > 99.9%; UniProtKB fasta file (release Sunday, June 23, 2019; totally 21,177 both reviewed and unreviewed entries of the *Oryctolagus cuniculus* taxon only). The concatenated decoy sequences were generated automatically by reversing the target sequences in the search engine interface. To identify proteins, trypsin as a specific protease was settled, and no more than two internal missed cleavages were allowed. The precursor mass tolerance was set to ±5.0 ppm, and the fragment mass tolerance was fitted to ±0.003 Da. Carbamidomethylation of C (+57.021464 Da), oxidation of M (+15.994915 Da), ubiquitination of K as the GG-tag (+114.042927 Da), and the long ubiquitination tag of K (+383.228102 Da) were established as variable modifications. Peptide spectrum matches (PSMs), peptides, and proteins were validated at a 1.0% false discovery rate (FDR) estimated using the decoy hit distribution and inferred from the spectrum identification results using PeptideShaker version 1.16.

## Ethics Statement

All experiments comply with the ARRIVE guidelines and were be carried out in accordance with the U.K. Animals (Scientific Procedures) Act, 1986 and associated guidelines, EU Directive 2010/63/EU for animal experiments, or the National Institutes of Health guide for the care and use of Laboratory animals (NIH Publications No. 8023, revised 1978).

All manipulations with animals were approved by the Animal Care and Use Committee at the Institute of Biomedical Chemistry (Moscow).

## CRediT Author Statement

**Olga Buneeva:** Formal analysis, Writing – original draft; **Arthur Kopylov:** Investigation; **Svetlana Kaloshina:** Formal analysis; **Victor Zgoda:** Formal analysis; **Alexei Medvedev:** Conceptualization, Writing – original draft, Writing – review & editing.

## Declaration of Competing Interest

The authors declare that they have no known competing financial interests or personal relationships which have or could be perceived to have influenced the work reported in this article.

## Appendix Supplementary materials

Image, application 1

Image, application 2
