# Supplementary material for: 20S and 26S proteasome-binding proteins of the rabbit brain: A proteomic dataset
Source: Data Brief. 2021 Aug 11;38:107276. doi: 10.1016/j.dib.2021.107276 (PMC8379623; doi:10.1016/j.dib.2021.107276)
Supplement: Supplementary file 1 [file mmc1.docx]

**Supplementary tables**

**Table 1. Proteins of 26S proteasome fraction (Rabbit brain)**

| No | Accession (Swiss-Prot) | Description | Gene name | Coverage | # Peptides | # Unique  Peptides | PSMs | Score | MW (kDa) |
| --- | --- | --- | --- | --- | --- | --- | --- | --- | --- |
| Regulatory particle subunits | | | | | | | | | |
| 1 | G1SVF2 | 26S proteasome non-ATPase regulatory subunit 1 | PSMD1 | 1,43 | 3 | 3 | 5 | Infinity | 108,431 |
| 2 | G1SSA2 | 26S proteasome non-ATPase regulatory subunit 2 | PSMD2 | 5,71 | 5 | 5 | 6 | Infinity | 115,167 |
| 3 | G1TP15 | Proteasome 26S subunit, non-ATPase 3 | PSMD3 | 6,02 | 8 | 8 | 8 | Infinity | 57,136 |
| 4 | B7NZD2 | Proteasome 26S subunit, non-ATPase, 4 (Predicted)  26S proteasome regulatory subunit RPN10 | PSMD4 | 2,89 | 2 | 2 | 2 | 27.530 | 41,061 |
| 5 | G1U115 | Proteasome 26S subunit, non-ATPase 6 | PSMD6 | 4,37 | 2 | 2 | 2 | 36.515 | 45,634 |
| 6 | G1SVT4 | Proteasome 26S subunit, non-ATPase 7 | PSMD7 | 5,24 | 2 | 2 | 2 | 41.597 | 44,375 |
| 7 | G1U354 | Proteasome 26S subunit, non-ATPase 8 | PSMD8 | 2,85 | 2 | 2 | 2 | Infinity | 39,601 |
| 8 | G1SL46 | 26S proteasome non-ATPase regulatory subunit 9 | N/A | 5,38 | 1 | 1 | 1 | Infinity | 24,765 |
| 9 | G1SVA3 | Proteasome 26S subunit, non-ATPase 11 | PSMD11 | 13,74 | 5 | 5 | 8 | Infinity | 47,434 |
| 10 | G1T6D4 | Proteasome 26S subunit, non-ATPase 12 | PSMD12 | 2,95 | 1 | 2 | 2 | Infinity | 51,295 |
| 11 | G1SM51 | Proteasome 26S subunit, non-ATPase 13 | PSMD13 | 4,79 | 4 | 4 | 4 | 50.579 | 42,741 |
| 12 | G1SFE0 | Proteasome 26S subunit, non-ATPase 14 | PSMD14 | 4,47 | 2 | 2 | 2 | Infinity | 32,663 |
| 13 | G1SYV0 | Proteasome 26S subunit, ATPase 2 | PSMC2 | 4,89 | 4 | 4 | 4 | 59.118 | 45,768 |
| 14 | G1TLQ8 | Proteasome 26S subunit, ATPase 3 | PSMC3 | 8,43 | 3 | 3 | 4 | Infinity | 49,133 |
| 15 | G1TUD6 | Proteasome 26S subunit, ATPase 4 | PSMC4 | 10,44 | 5 | 5 | 5 | Infinity | 55,811 |
| 16 | G1T3S1 | Proteasome 26S subunit, ATPase 6 | PSMC6 | 2.73 | 5 | 5 | 6 | 53.602 | 49,133 |
| Core particle subunits | | | | | | | | | |
| 1 | G1SDA8 | Proteasome subunit alpha type-1 | PSMA1 | 14,45 | 4 | 4 | 5 | Infinity | 28,609 |
| 2 | G1T2L1 | Proteasome subunit alpha type | N/A | 8,12 | 1 | 2 | 2 | Infinity | 25,899 |
| 3 | G1SZ14 | Proteasome subunit alpha type-3 | PSMA3 | 3,68 | 3 | 3 | 3 | Infinity | 36,284 |
| 4 | G1T519 | Proteasome subunit alpha type-4 | PSMA4 | 3,83 | 1 | 4 | 4 | 41,908 | 29,484 |
| 5 | G1T670 | Proteasome subunit alpha type-5 | PSMA5 | 5 | 1 | 1 | 1 | Infinity | 26,230 |
| 6 | G1T9V4 | Proteasome subunit alpha type-6 | PSMA6 | 9,35 | 2 | 2 | 2 | Infinity | 27,399 |
| 7 | G1SWI7 | Proteasome 20S subunit alpha 8 | PSMA8 | 8 | 2 | 2 | 2 | Infinity | 27,968 |
| 8 | G1SU71 | Proteasome subunit beta type-1 | PSMB1 | 4,15 | 1 | 1 | 1 | Infinity | 26,487 |
| 9 | G1T4X8 | Proteasome subunit beta type-2 | PSMB2 | 5,47 | 1 | 1 | 1 | Infinity | 22,82 |
| 10 | G1T4Q9 | Proteasome subunit beta type-5 | PSMB5 | 8,65 | 2 | 2 | 2 | Infinity | 28,764 |
| 11 | G1T235 | Proteasome subunit beta type-6 | PSMB6 | 8,79 | 2 | 2 | 2 | Infinity | 25,389 |
| Other components of UPS | | | | | | | | | |
| 1 | P62975 | Ubiquitin | UBIQ | 32,89 | 2 | 2 | 3 | Infinity | 8,565 |
| 2 | Q29504 | Ubiquitin-like modifier-activating enzyme 1 | UBA1 | 2,17 | 2 | 3 | 3 | 41.174 | 117,688 |
| 3 | G1TSL5 | Ubiquitin recognition factor in ER associated degradation 1 | UFD1 | 4,23 | 2 | 2 | 2 | 29.558 | 34,478 |
| 4 | G1THY3 | Ubiquitin conjugating enzyme E2 V1 | UBE2V1 | 6,76 | 2 | 2 | 2 | Infinity | 16,645 |
| 5 | G1TEW4 | Ubiquitin specific peptidase 9, X-linked | USP9X | 1,52 | 7 | 7 | 8 | Infinity | 292,129 |
| 6 | G1SK00 | Ubiquitinyl hydrolase 1 | USP5 | 7,72 | 12 | 12 | 16 | Infinity | 96,317 |
| 7 | G1SZR6 | Cullin 3 | CUL3 | 13,77 | 6 | 6 | 7 | Infinity | 89,183 |
| 8 | G1SN14 | Cullin associated and neddylation dissociated 1 | CAND1 | 7,13  6,88 | 13  10 | 13  10 | 14  11 | 34,208  Infinity | 133,529 |
| Metabolic enzymes | | | | | | | | | |
| 1 | **B7NZE9** | Creatine kinase, mitochondrial 1A (Predicted) | CKMT1A | 11,75 | 5 | 4 | 6 | Infinity | 46,992 |
| 2 | **P00563** | Creatine kinase M-type | CKM | 13,12 | 8 | 1 | 12 | Infinity | 43,085 |
| 3 | **P00567** | Creatine kinase B-type | CKB | 28,61 | 11 | 0 | 19 | Infinity | 42,636 |
| 4 | Q03505 | Alcohol dehydrogenase 1 | ADH1 | 19,73 | 8 | 8 | 29 | Infinity | 39,588 |
| 5 | **P13491** | L-lactate dehydrogenase A chain | LDHA | 3,61 | 1 | 0 | 1 | Infinity | 36,541 |
| 6 | **G1TYA7** | L-lactate dehydrogenase | LDHB | 26,59 | 10 | 10 | 11 | Infinity | 36,611 |
| 7 | **P11974** | Pyruvate kinase PKM | PKM | 11,86 | 9 | 7 | 10 | 126.435 | 58,011 |
| 8 | **P00939** | Triosephosphate isomerase | TPI1 | 31,85 | 8 | 8 | 10 | Infinity | 26,609 |
| 9 | **P00883** | Fructose-bisphosphate aldolase A | ALDOA | 24.73 | 11 | 10 | 15 | Infinity | 39,318 |
| 10 | **G1T652** | Fructose-bisphosphate aldolase | ALDOC | 17,58 | 7 | 7 | 9 | Infinity | 39,336 |
| 11 | **P00637** | Fructose-1,6-bisphosphatase 1 | FBP1 | 25,74 | 8 | 6 | 9 | Infinity | 36,554 |
| 12 | P00489 | Glycogen phosphorylase, muscle form | PYGM | 5.58 | 6 | 0 | 8 | Infinity | 97,228 |
| 13 | **P00511** | ATP-dependent 6-phosphofructokinase, muscle type | PFKM | 7,18 | 7 | 5 | 8 | Infinity | 85,149 |
| 14 | G1U7S4 | Phosphoglycerate mutase | PGAM2 | 8,3 | 4 | 0 | 4 | Infinity | 28,649 |
| 15 | G1TDS5 | Glutamate decarboxylase 2 | GAD2 | 3,25 | 3 | 3 | 3 | 37.673 | 65,321 |
| 16 | G1SQG5 | Malate dehydrogenase | MDH1 | 10.18 | 5 | 5 | 7 | 78.522 | 36,472 |
| 17 | G1T5L6 | Phosphodiesterase | NCKAP1L | 2.38 | 6 | 6 | 6 | Infinity | 190,880 |
| 18 | G1T1V5 | Carbonic anhydrase 3 | CA3 | 4,46 | 2 | 2 | 2 | 39.626 | 30,348 |
| 19 | G1SZ63 | Aldehyde dehydrogenase 9 family member A1 | ALDH9A1 | 7,08 | 3 | 2 | 3 | 44.663 | 46,439 |
| 20 | G1SIY9 | Aldehyde dehydrogenase 5 family member A1 | ALDH5A1 | 11,49 | 6 | 6 | 6 | Infinity | 55,951 |
| 21 | Q75NJ2 | Aldehyde dehydrogenase | aldDH | 24,4 | 27 | 24 | 33 | Infinity | 54,419 |
| 22 | **G1SNJ9** | Phosphoglycerate kinase | PGK2 | 9,83 | 4 | 0 | 4 | Infinity | 44,732 |
| 23 | **G1SYJ4** | Enolase 1 | ENO1 | 6,45 | 6 | 5 | 6 | Infinity | 47,243 |
| 24 | **G1SK04** | Enolase 2 | ENO2 | 8,76 | 3 | 2 | 5 | Infinity | 47,194 |
| 25 | **P46406** | Glyceraldehyde-3-phosphate dehydrogenase | GAPDH | 23,42 | 10 | 10 | 16 | Infinity | 35,757 |
| 26 | G1SPL1 | D-3-phosphoglycerate dehydrogenase | PHGDH | 5,07 | 3 | 2 | 3 | Infinity | 56,426 |
| 27 | G1T7Z0 | 6-phosphogluconate dehydrogenase, decarboxylating | PGD | 6,83 | 4 | 3 | 4 | Infinity | 69,893 |
| 28 | G1SI20 | Isocitrate dehydrogenase [NADP] | IDH1 | 25,54 | 11 | 8 | 11 | Infinity | 46,865 |
| 29 | P00949 | Phosphoglucomutase-1 | PGM1 | 16,37 | 6 | 6 | 6 | Infinity | 61,519 |
| 30 | G1TVT8 | Glycerol-3-phosphate dehydrogenase [NAD(+)] | GPD1 | 14,94 | 6 | 6 | 7 | Infinity | 37,283 |
| 31 | G1TA59 | Isocitrate dehydrogenase [NAD] subunit, mitochondrial | IDH3A | 11,75 | 5 | 5 | 5 | Infinity | 39,645 |
| 32 | Q95KM0 | Arginase-1 | ARG1 | 26,71 | 9 | 9 | 10 | Infinity | 34,427 |
| 33 | G1SG64 | Arginase | ARG1 | 20,41 | 9 | 9 | 10 | Infinity | 36,166 |
| 34 | G1SVB6 | Glutamate dehydrogenase | GLUD1 | 29,78 | 17 | 17 | 25 | Infinity | 49,489 |
| Components of cytoskeleton | | | | | | | | | |
| 1 | G1SNP7 | Microtubule associated protein 1A | MAP1A | 14,87 | 44 | 43 | 59 | Infinity | 313,541 |
| 2 | G1T892 | Microtubule associated protein RP/EB family member 3 | MAPRE3 | 24,56 | 8 | 6 | 11 | Infinity | 31,959 |
| 3 | G1SJF5 | Microtubule associated protein 6 | MAP6 | 18,7 | 12 | 12 | 13 | Infinity | 94,114 |
| 4 | G1SKS8 | Microtubule-associated protein | MAP4 | 1,38 | 4 | 4 | 4 | Infinity | 129,842 |
| 5 | G1TQR0 | Actinin alpha 1 | ACTN1 | 29,15 | 34 | 19 | 49 | Infinity | 102,946 |
| 6 | G1TUC8 | Actinin alpha 4 | ACTN4 | 24,06 | 22 | 9 | 31 | Infinity | 87,388 |
| 7 | P29751 | Actin, cytoplasmic 1 | ASTB | 51,2 | 25 | 4 | 98 | Infinity | 41,756 |
| 8 | G1SKS0 | Actin-related protein 2/3 complex subunit | ARPC1A | 6,49 | 3 | 3 | 5 | Infinity | 41,542 |
| 9 | G1T4C2 | Dynamin 1 | DNM1 | 43,83 | 26 | 20 | 46 | Infinity | 51,765 |
| 10 | G1T0B7 | Tubulin alpha chain | LOC100350027 | 30,53 | 37 | 0 | 126 | Infinity | 46,215 |
| 11 | G1TR82 | Tubulin alpha chain | TUBA4A | 59,6 | 63 | 7 | 284 | Infinity | 49,893 |
| 12 | G1SY02 | Tubulin beta chain | TUBB2A | 69,21 | 77 | 3 | 227 | 75,578 | 49,874 |
| 13 | G1SH05 | Tubulin beta chain | TUBB | 65,99 | 72 | 7 | 212 | Infinity | 49,694 |
| 14 | G1T346 | Spectrin alpha, non-erythrocytic 1 | SPTAN1 | 46,83 | 129 | 129 | 172 | Infinity | 289,345 |
| 15 | G1T7I5 | Spectrin beta chain | SPTBN2 | 13,44 | 36 | 30 | 43 | Infinity | 270,709 |
| 16 | G1SN11 | Spectrin beta chain | SPTBN1 | 19,42 | 69 | 61 | 98 | Infinity | 271,101 |
| 17 | G1SCI0 | Dynein cytoplasmic 1 heavy chain 1 | DYNC1H1 | 18,87 | 75 | 75 | 102 | Infinity | 378,292 |
| 18 | G1SWS9 | Vimentin | VIM | 14,38 | 11 | 8 | 11 | Infinity | 53,622 |
| 19 | G1T926 | Dynactin subunit 1 | DCTN1 | 9,84 | 16 | 16 | 16 | Infinity | 142,154 |
| 20 | G1T0Y9 | Dynactin subunit 2 | DCTN2 | 24,63 | 15 | 15 | 23 | Infinity | 44,209 |
| 21 | G1SD49 | Dynactin subunit 3 | DCTN3 | 23,66 | 6 | 6 | 6 | 101,394 | 21,162 |
| 22 | G1SRP7 | Importin subunit alpha | KPNA3 | 5,72 | 5 | 5 | 8 | Infinity | 61,848 |
| 23 | G1T868 | Clathrin light chain | CLTB | 12,39 | 3 | 3 | 3 | Infinity | 24,852 |
| 24 | G1TBL | Clathrin heavy chain | CLTC | 28,35 | 60 | 45 | 102 | Infinity | 216,956 |
| Proteins of signal transduction and trafficing | | | | | | | | | |
| 1 | G1SZD6 | 14-3-3 protein theta | YWHAQ | 49,8 | 17 | 11 | 43 | Infinity | 27,758 |
| 2 | **P62160** | Calmodulin | CALM | 18,79 | 4 | 4 | 4 | Infinity | 16,826 |
| 3 | Q6PNB6 | Guanine nucleotide-binding protein subunit beta-5 | GNB5 | 3,4 | 2 | 2 | 2 | Infinity | 38,731 |
| 4 | G1TFL3 | GTP-binding nuclear protein Ran |  | 6,06 | 1 | 1 | 2 | 27,580 | 18,884 |
| 5 | Q9N0Z6 | Sodium/potassium-transporting ATPase subunit alpha-1 | ATP1A1 | 4,69 | 5 | 3 | 5 | Infinity | 112,922 |
| 6 | Q9TT37 | Sodium/potassium-transporting ATPase subunit beta-1 | ATP1B1 | 7,26 | 4 | 4 | 4 | Infinity | 34,918 |
| 7 | G1SR29 | ATPase H+ transporting V1 subunit A | ATP6V1A | 33,08 | 17 | 17 | 23 | Infinity | 58,089 |
| 8 | G1SEJ4 | ATPase H+ transporting V1 subunit B2 | ATP6V1B2 | 38,16 | 22 | 22 | 29 | Infinity | 56,367 |
| 9 | G1SS33 | ATPase H+ transporting V1 subunit E1 | ATP6V1E1 | 32,74 | 9 | 9 | 16 | Infinity | 26,127 |
| 10 | G1SUT0 | Transportin 2 | TNPO2 | 2,22 | 4 | 0 | 5 | Infinity | 100,898 |
| 11 | G1TEH2 | V-type proton ATPase subunit C | ATP6V1C1 | 18,06 | 11 | 11 | 12 | Infinity | 43,946 |
| 12 | O97755 | V-type proton ATPase subunit D | ATP6V1D | 14,98 | 6 | 6 | 7 | Infinity | 28,235 |
| 13 | G1T239 | V-type proton ATPase subunit D1 | ATP6V0D1 | 6,84 | 2 | 2 | 2 | Infinity | 40,303 |
| 14 | G1T6S6 | V-type proton ATPase subunit F | ATP6V1F | 26,89 | 4 | 4 | 5 | Infinity | 13,336 |
| 15 | G1TZF3 | V-type proton ATPase subunit G | ATP6V1G2 | 13,92 | 2 | 2 | 2 | Infinity | 17,066 |
| 16 | G1TBC4 | V-type proton ATPase subunit H | ATP6V1H | 17,18 | 8 | 8 | 9 | Infinity | 55,886 |
| Protective proteins | | | | | | | | | |
| 1 | G1TQP4 | Heat shock protein family A (Hsp70) member 12A | HSPA12A | 8,67 | 10 | 10 | 11 | Infinity | 76,841 |
| 2 | **G1T3Y8** | Heat shock protein family D (Hsp60) member 1 | HSPD1 | 15,92 | 11 | 11 | 12 | Infinity | 56,632 |
| 3 | G1SMM5 | DnaJ heat shock protein family (Hsp40) member A2 | DNAJA2 | 7,52 | 3 | 3 | 5 | Infinity | 45,730 |
| 4 | P30947 | Heat shock protein HSP 90-beta | HSP90AB1 | 16,67 | 15 | 3 | 20 | Infinity | 83,415 |
| 5 | P30946 | Heat shock protein HSP 90-alpha | HSP90AA1 | 15,56 | 16 | 9 | 24 | Infinity | 79,683 |
| 6 | G1SYE2 | DnaJ heat shock protein family (Hsp40) member C6 | DNAJC6 | 7,49 | 6 | 6 | 7 | Infinity | 92,313 |
| 7 | G1T1V9 | Heat shock protein family A (Hsp70) member 2 | HSPA2 | 17,01 | 16 | 3 | 33 | 30,838 | 69,610 |
| 8 | G1T9M9 | Heat shock protein family A (Hsp70) member 8 | HSPA8 | 45,22 | 39 | 28 | 75 | Infinity | 71,038 |
| 9 | **P11909** | Glutathione peroxidase 1 | GPX1 | 34 | 4 | 4 | 5 | Infinity | 21,869 |
| 10 | G1T0R9 | Glutathione S-transferase | GSTM3 | 6,42 | 3 | 3 | 4 | Infinity | 25,481 |
| 11 | P46409 | Glutathione S-transferase Mu 1 | N/A | 20,18 | 7 | 8 | 9 | Infinity | 25,401 |
| 12 | O62648 | Sulfotransferase | N/A | 2,75 | 1 | 1 | 1 | Infinity | 33,561 |
| 13 | Q95KM3 | Sulfotransferase | N/A | 17,63 | 6 | 4 | 8 | Infinity | 34,171 |
| 14 | G1T6W7 | Catalase | CAT | 6,07 | 2 | 2 | 3 | Infinity | 59,619 |
| 15 | **G1SPZ7** | Glutathione peroxidase | N/A | 26,21 | 3 | 3 | 4 | Infinity | 16,167 |
| 16 | P08628 | Thioredoxin | TXN | 20,95 | 2 | 2 | 2 | Infinity | 11,752 |
| 17 | G1SRF7 | Heat shock protein family A (Hsp70) member 9 | HSPA9 | 5,01 | 3 | 3 | 3 | Infinity | 73,537 |
| 18 | G1U9S7 | T-complex protein 1 subunit alpha | TCP1 | 3,24 | 2 | 2 | 2 | 36,473 | 60,221 |
| 19 | G1U9T8 | T-complex protein 1 subunit delta | CCT4 | 5,88 | 3 | 3 | 4 | Infinity | 56,988 |
| 20 | G1SCN8 | T-complex protein 1 subunit gamma | CCT3 | 6,06 | 4 | 4 | 6 | Infinity | 60,550 |
| 21 | O77622 | T-complex protein 1 subunit zeta | CCT6 | 6,21 | 4 | 4 | 4 | 54,868 | 57,988 |
| Protein regulators of gene expression, cell division, and differentiation | | | | | | | | | |
| 1 | G1SK22 | 40S Ribosomal protein S27a | RPS27A | 30,13 | 6 | 6 | 21 | Infinity | 17,953 |
| 2 | G1TGI6 | 40S ribosomal protein SA | RPSA | 4,38 | 1 | 0 | 1 | Infinity | 33,115 |
| 3 | **P68105** | Elongation Factor 1-alpha 1 | EEF1A1 | 16,23 | 7 | 7 | 14 | Infinity | 50,109 |
| 4 | **P34826** | Elongation factor 1-beta | EEF1B | 4 | 2 | 2 | 3 | Infinity | 24,733 |
| 5 | **P29694** | Elongation factor 1-gamma | EEF1G | 18,99 | 10 | 10 | 15 | Infinity | 50,017 |
| 6 | **P53787** | Elongation factor 1-delta | EEF1D | 11,79 | 3 | 3 | 3 | Infinity | 31,055 |
| 7 | G1T336 | DEAD-box helicase 3, X-linked | DDX3X | 4,23 | 4 | 4 | 4 | Infinity | 73,108 |
| 8 | G1SQZ4 | RuvB-like helicase | RUVBL2 | 8,86 | 5 | 5 | 5 | Infinity | 51,125 |
| 9 | G1SMY1 | DEAD-box helicase 17 | DDX17 | 19,54 | 17 | 16 | 19 | Infinity | 72,310 |
| 10 | G1SMM7 | Small nuclear ribonucleoprotein D3 polypeptide | SNRPD3 | 7,94 | 2 | 2 | 2 | Infinity | 13,907 |
| 11 | G1SGB5 | Splicing factor proline and glutamine rich | SFPQ | 17,13 | 11 | 10 | 12 | Infinity | 57,332 |
| 12 | G1TWL0 | Heterogeneous nuclear ribonucleoprotein A2/B1 | HNRNPA2B1 | 25,21 | 12 | 9 | 14 | Infinity | 37,386 |
| 13 | G1SZ03 | Eukaryotic translation initiation factor 3 subunit B | EIF3B | 2,8 | 2 | 2 | 2 | Infinity | 78,591 |
| 14 | O77768 | Heterogeneous nuclear ribonucleoprotein C | HNRNPC | 12,75 | 7 | 7 | 7 | Infinity | 33,664 |
| 15 | G1SLT8 | Heterogeneous nuclear ribonucleoprotein H3 | HNRNPH3 | 5,49 | 2 | 2 | 3 | Infinity | 36,903 |
| 16 | G1TD41 | Heterogeneous nuclear ribonucleoprotein H1 | HNRNPH1 | 7,8 | 5 | 5 | 7 | Infinity | 49,198 |
| 17 | O19049 | Heterogeneous nuclear ribonucleoprotein K | HNRNPK | 29,16 | 17 | 17 | 31 | Infinity | 50,928 |
| 18 | **G1SN68** | Glutaminyl-tRNA synthetase | QARS | 6,19 | 4 | 4 | 4 | Infinity | 87,320 |
| 19 | **G1T846** | Aspartyl-tRNA synthetase | DARS | 6,99 | 6 | 6 | 7 | Infinity | 57,220 |
| 20 | **G1T2I4** | Glutamyl-prolyl-tRNA synthetase | EPRS | 3,64 | 10 | 10 | 10 | Infinity | 170,236 |
| 21 | **G1TWP4** | Valyl-tRNA synthetase | VARS | 2,86 | 5 | 5 | 6 | Infinity | 139,085 |

**Table 2. Proteins of 20S proteasome fraction (Rabbit brain)**

| No | Accession (Swiss-Prot) | Description | Gene name | Coverage | # Peptides | # Unique  Peptides | # PSMs | Score | MW (kDa) |
| --- | --- | --- | --- | --- | --- | --- | --- | --- | --- |
| Core particle subunits | | | | | | | | | |
| 1 | G1SDA8 | Proteasome subunit alpha type-1 | PSMA1 | 14,45 | 4 | 4 | 5 | Infinity | 28,609 |
| 2 | G1T2L1 | Proteasome subunit alpha type | *N/A* | 23,08 | 5 | 3 | 5 | Infinity | 25,899 |
| 3 | G1SZ14 | Proteasome subunit alpha type-3 | PSMA3 | 13,19 | 6 | 6 | 7 | Infinity | 36,284 |
| 4 | G1T519 | Proteasome subunit alpha type-4 | PSMA4 | 3,07 | 3 | 3 | 3 | 33.115 | 29,484 |
| 5 | G1T670 | Proteasome subunit alpha type-5 | PSMA5 | 5 | 1 | 1 | 1 | Infinity | 26,230 |
| 6 | G1T9V4 | Proteasome subunit alpha type-6 | PSMA6 | 13,01 | 3 | 3 | 3 | Infinity | 27,399 |
| 7 | G1SWI7 | Proteasome 20S subunit alpha 8 | PSMA8 | 4,4 | 2 | 2 | 3 | Infinity | 27,985 |
| 8 | G1SU71 | Proteasome subunit beta type-1 | PSMB1 | 7,88 | 3 | 3 | 3 | Infinity | 26,487 |
| 9 | G1T4X8 | Proteasome subunit beta type-2 | PSMB2 | 4,98 | 1 | 1 | 1 | Infinity | 22,808 |
| 10 | G1SHV9 | Proteasome subunit beta type-3 | PSMB3 | 15,12 | 2 | 2 | 2 | Infinity | 22,976 |
| 11 | G1T918 | Proteasome subunit beta type-4 | PSMB4 | 4,4 | 2 | 2 | 3 | Infinity | 28,657 |
| 12 | G1T4Q9 | Proteasome subunit beta type-5 | PSMB5 | 18,42 | 6 | 6 | 7 | Infinity | 28,78 |
| 13 | G1T235 | Proteasome subunit beta type-6 | PSMB6 | 8,79 | 3 | 3 | 3 | Infinity | 25,389 |
| 14 | G1SWK8 | Proteasome subunit beta type-7 | PSMB7 | 3,31 | 1 | 1 | 1 | 22,304 | 32,438 |
| Other components of UPS | | | | | | | | | |
| 1 | P62975 | Ubiquitin | UBIQ | 72,37 | 7 | 6 | 8 | Infinity | 8,565 |
| 2 | Q29504 | Ubiquitin-like modifier-activating enzyme 1 | UBA1 | 25,8 | 29 | 29 | 43 | Infinity | 117,688 |
| 3 | U3KNK3 | Ubiquitin conjugating enzyme E2 D2 | UBE2D2 | 7,48 | 1 | 1 | 2 | Infinity | 16,724 |
| 4 | G1U3G0 | Ubiquitin conjugating enzyme E2 O | UBE2O | 0.78 | 3 | 3 | 3 | 39.799 | 140,599 |
| 5 | G1THY3 | Ubiquitin conjugating enzyme E2 V1 | UBE2V1 | 19,59 | 3 | 1 | 4 | Infinity | 16,645 |
| 6 | G1SK00 | Ubiquitinyl hydrolase 1 | USP5 | 5,41 | 5 | 5 | 6 | Infinity | 96,317 |
| 7 | G1TIZ1 | Ubiquitin carboxyl-terminal hydrolase | UCHL1 | 19,73 | 6 | 6 | 8 | Infinity | 24,844 |
| 8 | P40826 | Ubiquitin carboxyl-terminal hydrolase 14 | USP14 | 2,64 | 1 | 1 | 1 | Infinity | 55,922 |
| 9 | G1U8C4 | Proteasome activator subunit 1 | PSME1 | 4,82 | 2 | 2 | 2 | Infinity | 28,642 |
| 10 | G1SN14 | Cullin associated and neddylation dissociated 1 | CAND1 | 9,04 | 14 | 12 | 15 | Infinity | 133,529 |
| 11 | P13019 | Bleomycin hydrolase (Fragment) | BLMH | 5,42 | 2 | 2 | 2 | Infinity | 32,579 |
| Metabolic enzymes | | | | | | | | | |
| 1 | **P11974** | Pyruvate kinase PKM | PKM | 54,8 | 2 | 0 | 116 | Infinity | 58,048 |
| 2 | **G1SQD3** | Pyruvate kinase | PKM | 54,24 | 49 | 0 | 102 | Infinity | 64,513 |
| 3 | **P00883** | Fructose-bisphosphate aldolase A | ALDOA | 57,14 | 41 | 34 | 72 | Infinity | 39,318 |
| 4 | **P79226** | Fructose-bisphosphate aldolase B | ALDOB | 14,01 | 6 | 5 | 6 | Infinity | 39,580 |
| 5 | **G1T652** | Fructose-bisphosphate aldolase | ALDOC | 51,92 | 41 | 34 | 84 | Infinity | 39,336 |
| 6 | **P13491** | L-lactate dehydrogenase A chain | LDHA | 39,76 | 24 | 18 | 41 | Infinity | 36,541 |
| 7 | **G1TZK5** | L-lactate dehydrogenase | LDHAL6B | 5,56 | 5 | 3 | 9 | 71.550 | 41,482 |
| 8 | **G1TYA7** | L-lactate dehydrogenase | LDHB | 30,21 | 23 | 19 | 50 | Infinity | 36,611 |
| 9 | **G1SYJ4** | Enolase 1 | ENO1 | 51,15 | 29 | 0 | 108 | Infinity | 47,243 |
| 10 | **G1T659** | Enolase-phosphatase E1 | ENOPH1 | 3,63 | 1 | 1 | 1 | Infinity | 36,287 |
| 11 | **G1SK04** | Enolase 2 | ENO2 | 61,29 | 44 | 37 | 131 | Infinity | 47,194 |
| 12 | **P00939** | Triosephosphate isomerase | TPI1 | 42,74 | 11 | 11 | 15 | Infinity | 26,609 |
| 13 | **P46406** | Glyceraldehyde-3-phosphate dehydrogenase | GAPDH | 33,93 | 19 | 19 | 36 | Infinity | 35,757 |
| 14 | G1T1V5 | Carbonic anhydrase 3 | CA3 | 17,1 | 4 | 0 | 4 | Infinity | 30,348 |
| 15 | **G1SVY8** | Creatine kinase B-type | CKB | 53,54 | 46 | 45 | 242 | Infinity | 42,712 |
| 16 | **P00563** | Creatine kinase M-type | CKM | 5,77 | 5 | 2 | 9 | Infinity | 43,085 |
| 17 | **G1SNJ9** | Phosphoglycerate kinase | PGK2 | 12,95 | 5 | 0 | 10 | Infinity | 44,732 |
| 18 | P00489 | Glycogen phosphorylase, muscle form | PYGM | 5,58 | 6 | 0 | 7 | Infinity | 97,228 |
| 19 | Q03505 | Alcohol dehydrogenase 1 | ADH1 | 34,93 | 21 | 18 | 24 | Infinity | 39,563 |
| 20 | O19053 | Alcohol dehydrogenase class-3 | ADH5 | 4,55 | 4 | 2 | 5 | Infinity | 39,570 |
| 21 | G1U460 | Aldehyde dehydrogenase 6 family member A1 | ALDH6A1 | 4,06 | 2 | 2 | 2 | Infinity | 61,489 |
| 22 | G1SZ63 | Aldehyde dehydrogenase 9 family member A1 | ALDH9A1 | 13,68 | 10 | 9 | 13 | Infinity | 46,439 |
| 23 | **P00511** | ATP-dependent 6-phosphofructokinase, muscle type | PFKM | 21,15 | 22 | 0 | 32 | Infinity | 85,149 |
| 24 | G1TVT8 | Glycerol-3-phosphate dehydrogenase [NAD(+)] | GPD1 | 7,47 | 4 | 4 | 6 | Infinity | 37,283 |
| 25 | G1SYK0 | Glycerol-3-phosphate dehydrogenase [NAD(+)] | GPD1L | 3,59 | 1 | 0 | 1 | Infinity | 36,813 |
| 26 | G1T765 | Malate dehydrogenase | MDH2 | 49,7 | 18 | 18 | 41 | Infinity | 35,463 |
| 27 | G1SQG5 | Malate dehydrogenase | MDH1 | 34,43 | 17 | 17 | 34 | Infinity | 36,472 |
| 28 | **B7NZE9** | Creatine kinase, mitochondrial 1A (Predicted) | CKMT1A | 7,43 | 8 | 6 | 11 | Infinity | 46,99 |
| 29 | G1T7Z0 | 6-phosphogluconate dehydrogenase, decarboxylating | PGD | 6,06 | 5 | 5 | 5 | Infinity | 69,89 |
| 30 | G1SI20 | Isocitrate dehydrogenase [NADP] | IDH1 | 10,36 | 10 | 9 | 13 | Infinity | 46,87 |
| 31 | P00949 | Phosphoglucomutase-1 | PGM1 | 18,68 | 15 | 15 | 22 | Infinity | 61,52 |
| 32 | G1TA59 | Isocitrate dehydrogenase [NAD] subunit, mitochondrial | IDH3A | 6,28 | 2 | 2 | 2 | Infinity | 39,65 |
| 33 | G1SVB6 | Glutamate dehydrogenase | GLUD1 | 20,67 | 13 | 13 | 18 | Infinity | 49,49 |
| 34 | G1T545 | Lactoylglutathione lyase | GLO1 | 32,09 | 4 | 4 | 4 | Infinity | 20,934 |
| 35 | G1TC46 | S-adenosylmethionine synthase | MAT1A | 7,52 | 3 | 1 | 3 | Infinity | 43,917 |
| 36 | G1SHD6 | S-adenosylmethionine synthase | MAT2A | 9,15 | 5 | 3 | 5 | Infinity | 36,306 |
| Components of cytoskeleton | | | | | | | | | |
| 1 | G1T892 | Microtubule associated protein RP/EB family member 3 | MAPRE3 | 3,2 | 1 | 0 | 1 | Infinity | 31,96 |
| 2 | G1SNP7 | Microtubule associated protein 1A | MAP1A | 8,07 | 26 | 25 | 32 | Infinity | 313,54 |
| 3 | G1TR82 | Tubulin alpha chain | TUBA4A | 52,68 | 37 | 4 | 104 | Infinity | 49,89 |
| 4 | G1T0B7 | Tubulin alpha chain | *N/A* | 29,33 | 25 | 0 | 63 | Infinity | 46,22 |
| 5 | G1SH05 | Tubulin beta chain | TUBB | 53,83 | 50 | 4 | 128 | Infinity | 49,69 |
| 6 | G1SKS0 | Actin-related protein 2/3 complex subunit | ARPC1A | 11,08 | 5 | 5 | 6 | Infinity |  |
| 7 | G1SY02 | Tubulin beta chain | TUBB2B | 58,43 | 57 | 2 | 140 | Infinity | 49,87 |
| 8 | P29751 | Actin, cytoplasmic 1 | ACTB | 30,4 | 22 | 3 | 47 | Infinity | 41,73 |
| 9 | G1T346 | Spectrin alpha, non-erythrocytic 1 | SPTAN1 | 0,36 | 3 | 3 | 3 | Infinity | 289,345 |
| 10 | G1SN11 | Spectrin beta chain | SPTBN1 | 1,71 | 7 | 7 | 8 | Infinity | 271,101 |
| Proteins of signal transduction and trafficking | | | | | | | | | |
| 1 | G1TRY5 | Plastin 3 | PLS3 | 3,17 | 4 | 4 | 4 | 38,558 | 70,794 |
| 2 | G1TBL6 | Clathrin heavy chain | CLTC | 0,95 | 2 | 2 | 2 | 18,511 | 216,956 |
| 3 | Q6Q6X0 | 14-3-3 protein theta | YWHAQ | 24,9 | 8 | 3 | 13 | Infinity | 27,760 |
| 4 | G1SEJ4 | ATPase H+ transporting V1 subunit B2 | ATP6V1B2 | 3,72 | 3 | 3 | 3 | 41,782 | 56,367 |
| 5 | P62160 | Calmodulin | CALM | 40,94 | 10 | 10 | 17 | Infinity | 16,826 |
| Protective proteins | | | | | | | | | |
| 1 | G1TQP4 | Heat shock protein family A (Hsp70) member 12A | HSPA12A | 22,83 | 19 | 17 | 34 | Infinity | 76,84 |
| 2 | G1T1V9 | Heat shock protein family A (Hsp70) member 2 | HSPA2 | 19,21 | 21 | 4 | 41 | 111,88 | 69,61 |
| 3 | G1T9M9 | Heat shock protein family A (Hsp70) member 8 | HSPA8 | 43,06 | 42 | 27 | 83 | Infinity | 71,04 |
| 4 | G1U0U5 | Heat shock protein family A (Hsp70) member 4 like | HSPA4L | 13,66 | 18 | 14 | 25 | Infinity | 107,085 |
| 5 | G1SV05 | Heat shock protein family A (Hsp70) member 4 | HSPA4 | 18,1 | 17 | 14 | 24 | Infinity | 83,015 |
| 6 | G1U7L4 | Heat shock protein family A (Hsp70) member 5 | HSPA5 | 25,65 | 23 | 19 | 34 | Infinity | 72,391 |
| 7 | G1SRF7 | Heat shock protein family A (Hsp70) member 9 | HSPA9 | 2,06 | 4 | 3 | 4 | Infinity | 73,537 |
| 8 | G1T840 | Heat shock protein family H (Hsp110) member 1 | HSPH1 | 24,45 | 27 | 21 | 41 | Infinity | 96,794 |
| 9 | **G1T3Y8** | Heat shock protein family D (Hsp60) member 1 | HSPD1 | 18,73 | 13 | 13 | 18 | Infinity | 56,63 |
| 10 | P30947 | Heat shock protein HSP 90-beta | HSP90AB1 | 38,02 | 44 | 10 | 102 | Infinity | 83,42 |
| 11 | P30946 | Heat shock protein HSP 90-alpha | HSP90AA1 | 40,78 | 44 | 28 | 107 | Infinity | 79,68 |
| 12 | **G1T8Z0** | Peroxiredoxin 6 | PRDX6 | 41,41 | 12 | 12 | 18 | Infinity | 25,233 |
| 13 | P08628 | Thioredoxin | TXN | 20,95 | 2 | 2 | 4 | Infinity | 11,753 |
| 14 | G1U3B8 | Thioredoxin like 1 | TXNL1 | 17,3 | 4 | 4 | 4 | Infinity | 32,243 |
| 15 | G1TV91 | Glutathione S-transferase | GSTM2 | 29,91 | 12 | 3 | 18 | Infinity | 26,00 |
| 16 | **P11909** | Glutathione peroxidase 1 | GPX1 | 6 | 2 | 2 | 2 | Infinity | 21,87 |
| 17 | P46409 | Glutathione S-transferase Mu 1 | *N/A* | 57,34 | 19 | 10 | 29 | Infinity | 25,4 |
| 18 | G1T0R9 | Glutathione S-transferase | LOC100357 | 57,34 | 18 | 6 | 29 | Infinity | 25,48 |
| 19 | G1SKS9 | Thioredoxin reductase 1, cytoplasmic | TXNRD1 | 4,88 | 6 | 6 | 8 | Infinity | 71,369 |
| 20 | G1U9S7 | T-complex protein 1 subunit alpha | TCP1 | 25,36 | 15 | 14 | 18 | Infinity | 60,221 |
| 21 | G1U9T8 | T-complex protein 1 subunit delta | CCT4 | 13,28 | 10 | 9 | 12 | Infinity | 56,99 |
| 22 | G1U9T1 | T-complex protein 1 subunit eta | CCT7 | 14 | 9 | 9 | 13 | Infinity | 59,421 |
| 23 | G1SCN8 | T-complex protein 1 subunit gamma | CCT3 | 17,8 | 13 | 13 | 18 | Infinity | 60,550 |
| 24 | O77622 | T-complex protein 1 subunit zeta (TCPZ_RABIT) | CCT6 | 20,9 | 13 | 13 | 14 | Infinity | 57,988 |
| 25 | G1TBC1 | Endoplasmin | HSP90B1 | 12,26 | 14 | 11 | 18 | Infinity | 89,259 |
| Protein regulators of gene expression, cell division, and differentiation | | | | | | | | | |
| 1 | G1U6N2 | 60S acidic ribosomal protein P0 | *N/A* | 2,97 | 1 | 2 | 3 | Infinity | 33,212457 |
| 2 | Q0QEW4 | Ribosomal protein L18 (Fragment) | RPL18 | 7,88 | 1 | 1 | 1 | Infinity | 18,685353 |
| 3 | G1SMR7 | Ribosomal protein L12 | RPL12 | 4,62 | 1 | 1 | 1 | 32,531 | 21,244 |
| 4 | G1TG04 | 60S ribosomal protein L6 | *N/A* | 3,44 | 1 | 1 | 1 | Infinity | 32,951726 |
| 5 | G1SK22 | Ribosomal protein S27a | RPS27A | 24,36 | 7 | 7 | 18 | Infinity | 17,953 |
| 6 | **P68105** | Elongation Factor 1-alpha 1 | EEF1A1 | 11,69 | 5 | 6 | 6 | Infinity | 50,109 |
| 7 | Q71V39 | Elongation factor 1-alpha 2 | EEF1A2 | 17,49 | 16 | 2 | 21 | Infinity | 50,438 |
| 8 | P34826 | Elongation factor 1-beta | EEF1B | 10,67 | 4 | 4 | 4 | Infinity | 24,733 |
| 9 | P29694 | Elongation factor 1-gamma | EEF1G | 17,85 | 10 | 10 | 16 | Infinity | 50,107 |
| 10 | G1SS73 | DEAD-box helicase 1 | DDX1 | 3,92 | 7 | 7 | 11 | Infinity | 82,387 |
| 11 | G1SMY1 | DEAD-box helicase 17 | DDX17 | 3,69 | 4 | 4 | 4 | Infinity | 72,310 |
| 12 | G1U7I9 | DExD-box helicase 39B | DDX39B | 4,98 | 5 | 5 | 6 | Infinity | 50,501 |
| 13 | G1TXI1 | Nucleophosmin | NPM1 | 5,46 | 1 | 0 | 1 | Infinity | 26,290 |
| 14 | G1SZL5 | E74 like ETS transcription factor 1 | ELF1 | 1,05 | 4 | 4 | 4 | Infinity | 105,799 |
| 15 | G1SYN3 | Leucine zipper transcription factor like 1 | LZTFL1 | 3,68 | 2 | 2 | 2 | Infinity | 34,724 |
| 16 | G1TC61 | LUC7 like 2, pre-mRNA splicing factor | LUC7L2 | 3,88 | 1 | 1 | 1 | Infinity | 45,516 |
| 17 | G1U194 | LUC7 like 3 pre-mRNA splicing factor | LUC7L3 | 4,18 | 1 | 1 | 1 | Infinity | 51,336 |
| 18 | G1TPN3 | Heterogeneous nuclear ribonucleoprotein A/B | HNRNPAB | 13,65 | 4 | 3 | 6 | Infinity | 30,304 |
| 19 | G1TWL0 | Heterogeneous nuclear ribonucleoprotein A2/B1 | HNRNPA2B1 | 4,53 | 2 | 0 | 2 | Infinity | 37,386 |
| 20 | O19049 | Heterogeneous nuclear ribonucleoprotein K | HNRNPK | 10,58 | 4 | 4 | 6 | Infinity | 50,928 |
| 21 | G1T7H0 | Heterogeneous nuclear ribonucleoprotein U | HNRNPU | 1,15 | 2 | 2 | 2 | 28,170 | 95,480 |
| 22 | G1SMM7 | Small nuclear ribonucleoprotein D3 polypeptide | SNRPD3 | 7,94 | 1 | 1 | 2 | 32,507 | 13,907 |
| 23 | G1TVT0 | Nucleolin | NCL | 4,74 | 8 | 8 | 10 | Infinity | 83,338 |
| 24 | G1SNT1 | Eukaryotic translation initiation factor 3 subunit J | EIF3J | 4,25 | 1 | 1 | 1 | Infinity | 29,400 |
| 25 | G1TE76 | Eukaryotic translation initiation factor 4H | EIF4H | 5,26 | 2 | 2 | 2 | Infinity | 27,369 |
| 26 | P29338 | Eukaryotic translation initiation factor 4E | EIF4E | 6,45 | 1 | 1 | 2 | Infinity | 25,033 |
| 27 | P10160 | Eukaryotic translation initiation factor 5A-1 | EIF5A | 12,99 | 2 | 0 | 4 | Infinity | 16,805 |
| 28 | G1SRA8 | Eukaryotic translation initiation factor 2 subunit 3 | EIF2S3 | 2,04 | 2 | 2 | 2 | Infinity | 53,459 |
| 29 | **G1SNM1** | Glycyl-tRNA synthetase | GARS | 8,18 | 8 | 8 | 12 | Infinity | 77,408 |
| 30 | **G1SDU6** | Threonyl-tRNA synthetase | TARS | 2,9 | 3 | 3 | 4 | 43,579 | 83,417 |
| 31 | **G1SW24** | Alanyl-tRNA synthetase | AARS | 8,58 | 8 | 8 | 9 | Infinity | 108,971 |
| 32 | **G1STW7** | Phenylalanyl-tRNA synthetase beta subunit | FARSB | 12,26 | 13 | 13 | 18 | Infinity | 64,632 |
| 33 | **G1TF82** | Alanyl-tRNA synthetase domain containing 1 | AARSD1 | 5,1 | 3 | 3 | 3 | 48,360 | 45,179 |
| 34 | **G1SIV7** | Asparaginyl-tRNA synthetase | NARS | 2,19 | 1 | 1 | 1 | 23,617 | 63,324 |
| 35 | **G1T846** | Aspartyl-tRNA synthetase | DARS | 2,99 | 3 | 3 | 3 | Infinity | 57,220 |
| 36 | **G1SLD6** | Histidyl-tRNA synthetase | HARS | 6,09 | 5 | 5 | 5 | Infinity | 57,435 |
| 37 | **G1T2I4** | Glutamyl-prolyl-tRNA synthetase | EPRS | 2,12 | 4 | 4 | 4 | Infinity | 170,236 |
