# Supplementary material for: 20S and 26S proteasome-binding proteins of the rabbit brain: A proteomic dataset
Source: Data Brief. 2021 Aug 11;38:107276. doi: 10.1016/j.dib.2021.107276 (PMC8379623; doi:10.1016/j.dib.2021.107276)
Supplement: Supplementary file 2 [file mmc2.docx]

**Table 3. Proteins of 26S proteasome fraction (Rabbit brain) identified in two independent experiments**

|  | Main Accession | Description | Gene Name | MW [kDa] | Confident Coverage [%] | #Peptides | #Unique | #PSMs | Score |
| --- | --- | --- | --- | --- | --- | --- | --- | --- | --- |
| Regulatory particle subunits | | | | | | | | | |
| 1 | G1SQL0 | AAA domain-containing protein | PSMC1 | 49,15 | 2,73 | 1 | 1 | 1 | Infinity |
| 2 | G1SLK2 | AAA domain-containing protein | PSMC5 | 45,6 | 8,37 | 4 | 4 | 4 | Infinity |
| Other components of UPS | | | | | | | | | |
| 1 | G1U101 | HECT domain E3 ubiquitin protein ligase 4 | HECTD4 | 484,53 | 0,23 | 4 | 4 | 4 | 35,69 |
| 2 | Q4PLJ0 | NEDD8 | NEDD8 | 9,07 | 11,11 | 1 | 1 | 1 | 23,6 |
| 3 | G1TDD2 | UBX domain protein 1 | UBXN1 | 33,16 | 4,04 | 1 | 1 | 2 | Infinity |
| Metabolic enzymes | | | | | | | | | |
| 1 | G1TVW5 | GDP-mannose pyrophosphorylase A | GMPPA | 45,17 | 3,41 | 3 | 3 | 3 | 31,56 |
| 2 | G1T489 | Acid phosphatase 1 | ACP1 | 18,74 | 5,45 | 1 | 1 | 2 | 27,64 |
| 3 | G1SN09 | GDP-mannose pyrophosphorylase B | GMPPB | 39,75 | 5,83 | 2 | 2 | 2 | 31,88 |
| 4 | G1U723 | 3alpha/17beta/20alpha-hydroxysteroid dehydrogenase | PGER5 | 36,62 | 2,48 | 7 | 2 | 7 | 42,59 |
| 5 | G1SRI8 | Hexokinase 1 | HK1 | 102,73 | 9,23 | 9 | 7 | 9 | Infinity |
| 6 | G1SSV4 | Phosphoinositide phospholipase C | PLCB1 | 123,23 | 0,83 | 1 | 1 | 1 | 23,6 |
| 7 | G1TSL1 | Isocitrate dehydrogenase [NAD] subunit, mitochondrial | IDH3B | 42,21 | 2,34 | 2 | 2 | 2 | 22 |
| 8 | G1TKQ5 | Pterin-4 alpha-carbinolamine dehydratase 1 | PCBD1 | 11,99 | 7,69 | 1 | 1 | 2 | 20,8 |
| 9 | P12345 | Aspartate aminotransferase, mitochondrial | GOT2 | 47,38 | 2,79 | 2 | 2 | 2 | Infinity |
| 10 | G1T704 | Sorbitol dehydrogenase | SORD | 38,14 | 2,51 | 1 | 1 | 1 | Infinity |
| 11 | G1TTJ1 | Dehydrogenase/reductase 11 | DHRS11 | 28,17 | 8,88 | 3 | 3 | 3 | Infinity |
| 12 | G1TK53 | N-acyl-L-amino-acid amidohydrolase | ACY1 | 53,51 | 4,13 | 2 | 2 | 2 | 47,2 |
| 13 | G1T9S4 | Acetyltransferase component of pyruvate dehydrogenase complex | DLAT | 68,162 | 1,24 | 2 | 2 | 2 | 32,7 |
| 14 | G1T3T2 | Glutamate decarboxylase 1 | GAD1 | 66,83 | 1,52 | 2 | 2 | 2 | 26,6 |
| 15 | G1T024 | Phosphoribosylformylglycinamidine synthase | PFAS | 143,52 | 0,75 | 3 | 3 | 3 | 28,5 |
| 16 | G1SXT1 | Adenosylhomocysteinase | AHCYL1 | 61,87 | 2,14 | 2 | 0 | 2 | Infinity |
| 17 | G1TWQ3 | NAD-dependent protein deacetylase | SIRT2 | 43,02 | 5,91 | 2 | 2 | 2 | Infinity |
| 18 | G1SL40 | Protein quaking | QKI | 37,45 | 4,12 | 1 | 1 | 1 | Infinity |
| 19 | G1T9T5 | Dihydrolipoamide S-succinyltransferase | DLST | 48,55 | 10,67 | 4 | 4 | 5 | Infinity |
| 20 | G1T837 | Peptidyl arginine deiminase 2 | PADI2 | 75,29 | 1,5 | 5 | 5 | 5 | 36,55 |
| 21 | G1U9T2 | Serine/threonine-protein phosphatase | PPP3CA | 58,65 | 20,15 | 16 | 7 | 27 | Infinity |
| 22 | G1T4H3 | Protein disulfide isomerase family A member 6 | PDIA6 | 47,97 | 3,18 | 1 | 1 | 2 | Infinity |
| 23 | G1TPI0 | Folate hydrolase 1 | FOLH1 | 84,77 | 1,19 | 3 | 3 | 3 | Infinity |
| 24 | G1TDC3 | Gal_mutarotas_2 domain-containing protein | GANAB | 109,38 | 7,35 | 8 | 8 | 8 | Infinity |
| 25 | G1SRB6 | Peptidylprolyl isomerase D | PPID | 41,04 | 4,86 | 2 | 2 | 2 | 34,1 |
| 26 | G1TRZ8 | Serine/threonine-protein phosphatase | PPP3CB | 59,08 | 20,38 | 17 | 8 | 26 | Infinity |
| 27 | G1TS42 | Glycogen debranching enzyme | AGL | 175,01 | 5,81 | 11 | 11 | 12 | Infinity |
| 28 | G1T7Z6 | Phosphoglycerate kinase | PGK1 | 44,68 | 8,13 | 5 | 0 | 5 | Infinity |
| 29 | G1T3P1 | Acyl-CoA thioesterase 7 | ACOT7 | 37,12 | 3,27 | 1 | 0 | 1 | 23,5 |
| 30 | G1SUM6 | Carboxypeptidase E | CPE | 52,86 | 4,45 | 2 | 2 | 2 | Infinity |
| 31 | O19132 | Nitric oxide synthase, brain | NOS1 | 160,76 | 0,7 | 2 | 2 | 2 | 30,6 |
| Components of cytoskeleton | | | | | | | | | |
| 1 | G1T5E6 | Sorting nexin 2 | SNX2 | 58,38 | 4,82 | 3 | 3 | 3 | Infinity |
| 2 | B7NZG7 | Sorting nexin 3 (Predicted) | SNX3 | 18,75 | 5,56 | 1 | 1 | 1 | 23,5 |
| 3 | G1T8C8 | Sorting nexin 12 | SNX12 | 19,16 | 5,42 | 1 | 1 | 2 | 27,53 |
| 4 | G1SFE4 | Synaptojanin-1 | SYNJ1 | 170,67 | 9,79 | 20 | 20 | 28 | Infinity |
| 5 | G1TJ20 | Dynamin-type G domain-containing protein | N/A | 74,81 | 10,86 | 9 | 8 | 11 | Infinity |
| 6 | G1T2V1 | Nebulette | NEBL | 116,69 | 1,08 | 2 | 2 | 2 | 25,69 |
| 7 | G1T4G9 | Tropomodulin 2 | TMOD2 | 39,43 | 5,98 | 2 | 2 | 2 | 33,5 |
| 8 | G1THX0 | Myelin basic protein | MBP | 32,75 | 25,5 | 8 | 8 | 10 | Infinity |
| 9 | G1SYD6 | Lamin A/C | LMNA | 74,02 | 3,17 | 3 | 3 | 3 | 69,7 |
| 10 | G1SY84 | Dynein cytoplasmic 1 intermediate chain 2 | DYNC1I2 | 67,56 | 3,48 | 1 | 1 | 1 | Infinity |
| 11 | P63169 | Dynein light chain 1, cytoplasmic | DYNLL1 | 10,36 | 12,36 | 2 | 2 | 4 | Infinity |
| 12 | G1TVQ3 | Dynein light chain roadblock | DYNLRB1 | 10,98 | 12,5 | 2 | 2 | 2 | 27,11 |
| 13 | G1U949 | Microtubule associated protein RP/EB family member 1 | MAPRE1 | 30 | 10,07 | 3 | 2 | 3 | Infinity |
| 14 | G1TDK0 | Myosin light chain 6 | MYL6 | 16,95 | 5,96 | 2 | 2 | 2 | 31,44 |
| 15 | P47789 | Myelin proteolipid protei | PLP1 | 30,07 | 3,97 | 1 | 1 | 1 | 27,69 |
| 16 | G1U6H6 | Four and a half LIM domains 1 | FHL1 | 33,54 | 4,05 | 2 | 2 | 2 | Infinity |
| 17 | G1SIT3 | WD repeat domain 47 | WDR47 | 110,02 | 1,01 | 3 | 3 | 3 | 31,57 |
| 18 | G1TQJ1 | Dematin actin binding protein | DMTN | 45,42 | 5,2 | 2 | 2 | 2 | Infinity |
| 19 | G1U9R8 | Gelsolin | GSN | 80,52 | 1,5 | 2 | 2 | 3 | Infinity |
| 20 | G1SCE4 | Clathrin light chain | CLTA | 27,09 | 10,48 | 3 | 3 | 3 | 52,19 |
| 21 | G1TW04 | Drebrin 1 | DBN1 | 75,21 | 3,47 | 3 | 3 | 4 | Infinity |
| 22 | G1T5J8 | Dynactin subunit 4 | DCTN4 | 52,4 | 7,39 | 3 | 3 | 3 | Infinity |
| 23 | G1STJ8 | Myosin VA | MYO5A | 217,91 | 0,48 | 2 | 2 | 2 | 26 |
| 24 | G1TFC2 | Tubulin alpha 8 | TUBA8 | 96,87 | 20,23 | 42 | 0 | 157 | 27,64 |
| 25 | G1TAM3 | Tubulin folding cofactor B | TBCB | 27,4 | 10,66 | 3 | 3 | 3 | 66,54 |
| 26 | G1SQY8 | Nuclear distribution C, dynein complex regulator | NUDC | 38,31 | 3,31 | 2 | 2 | 2 | 27,84 |
| 27 | G1TTN7 | Kinesin-like protein | KIF2A | 81,03 | 4,18 | 5 | 5 | 6 | Infinity |
| 28 | G1SVG8 | Oligodendrocyte myelin glycoprotein | OMG | 49,78 | 4,74 | 2 | 2 | 3 | 41 |
| 29 | G1SQR6 | Calponin | CNN3 | 36,32 | 17,93 | 6 | 6 | 7 | Infinity |
| 30 | G1SMS3 | ARP2 actin related protein 2 homolog | ACTR2 | 44,73 | 3,05 | 4 | 4 | 4 | Infinity |
| 31 | Q28626 | Alpha-1-syntrophin | SNTA1 | 53,73 | 4,16 | 4 | 4 | 4 | 54,1 |
| 32 | G1SDC1 | ARP3 actin related protein 3 homolog B | ACTR3B | 47,55 | 5,5 | 3 | 1 | 4 | 29,8 |
| 33 | G1T277 | Actin-related protein 2/3 complex subunit 3 | ARPC3 | 20,55 | 6,18 | 1 | 1 | 2 | 20,8 |
| Proteins of signal transduction and trafficing | | | | | | | | | |
| 1 | P20647 | Sarcoplasmic/endoplasmic reticulum calcium ATPase 2 | ATP2A2 | 114,63 | 2,02 | 4 | 4 | 4 | Infinity |
| 2 | G1TN33 | Septin-8 | SEPT8 | 62,41 | 3,5 | 6 | 3 | 10 | 45,26 |
| 3 | G1SXV1 | Septin 4 | SEPT4 | 55,32 | 11,3 | 6 | 6 | 9 | 125,25 |
| 4 | Q6Q6X0 | 14-3-3 protein theta | YWHAQ | 27,76 | 24,9 | 8 | 0 | 13 | Infinity |
| 5 | G1SJV2 | Ras related GTP binding A | RRAGA | 36,54 | 4,15 | 2 | 2 | 2 | Infinity |
| 6 | O97572 | Ras-related protein Rab-7a | RAB7A | 23,53 | 12,08 | 3 | 0 | 3 | 59,31 |
| 7 | G1SNP8 | Trafficking from ER to golgi regulator | TFG | 43,45 | 4,73 | 2 | 2 | 2 | Infinity |
| 8 | G1SLA4 | Protein lin-7 homolog | LIN7A | 25,98 | 15,88 | 4 | 0 | 4 | 29,57 |
| 9 | G1SY99 | Adaptor related protein complex 3 mu 2 subunit | AP3M2 | 46,9 | 4,78 | 2 | 2 | 3 | 42,51 |
| 10 | G1TE17 | Polypyrimidine tract binding protein 2 | PTBP2 | 58,09 | 2,05 | 3 | 3 | 3 | 21,7 |
| 11 | P80912 | Histidine triad nucleotide-binding protein 1 | HINT1 | 13,68 | 0 | 2 | 2 | 2 | Infinity |
| 12 | G1T5T2 | WD repeat domain 44 | WDR44 | 101,18 | 1,32 | 3 | 3 | 3 | 35,4 |
| 13 | G1SLS3 | AP-1 complex subunit gamma | AP1G1 | 91,28 | 1,22 | 2 | 2 | 2 | 25,86 |
| 14 | G1SDA4 | Coatomer subunit beta | COPB1 | 106,97 | 1,05 | 2 | 2 | 2 | 29,26 |
| 15 | G1T593 | Coatomer protein complex subunit zeta 1 | COPZ1 | 20,13 | 6,21 | 1 | 1 | 1 | 23,5 |
| 16 | G1TS36 | Membrane palmitoylated protein 6 | MPP6 | 62,65 | 5,05 | 10 | 9 | 10 | Infinity |
| 17 | G1SG68 | Importin subunit alpha | KPNA4 | 57,86 | 1,73 | 1 | 0 | 2 | Infinity |
| 18 | G1SJC7 | G3BP stress granule assembly factor 2 | G3BP2 | 54,09 | 2,7 | 1 | 1 | 1 | Infinity |
| 19 | G1T4G4 | Neuronal pentraxin 1 | NPTX1 | 37,12 | 3,26 | 1 | 1 | 1 | Infinity |
| 20 | G1TED6 | Annexin | ANXA5 | 35,92 | 5,61 | 2 | 2 | 2 | 36,2 |
| 21 | G1SF06 | EH domain containing 3 | EHD3 | 60,9 | 3,54 | 3 | 0 | 4 | Infinity |
| 22 | G1SCT5 | Coronin | N/A | 105,86 | 2,13 | 2 | 2 | 2 | 35,4 |
| 23 | G1U797 | FMR1 autosomal homolog 1 | FXR1 | 88,05 | 2,02 | 3 | 3 | 3 | Infinity |
| 24 | G1TCM5 | Protocadherin 1 | PCDH1 | 133,58 | 0,65 | 2 | 2 | 5 | 33,11 |
| 25 | G1SJX0 | WAS protein family member 3 | WASF3 | 54,97 | 5,01 | 2 | 2 | 3 | Infinity |
| 26 | G1SQI4 | Neural cell adhesion molecule 1 | NCAM1 | 96,17 | 6,15 | 7 | 7 | 7 | 114,51 |
| 27 | G1TUP2 | IRG-type G domain-containing protein | N/A | 47,4 | 3,43 | 1 | 1 | 1 | Infinity |
| 28 | G1T286 | Vacuolar protein sorting-associated protein 29 | VPS29 | 19,81 | 10,23 | 2 | 2 | 3 | Infinity |
| 29 | G1SFD5 | Epsin 2 | EPN2 | 54,18 | 2,19 | 1 | 1 | 1 | 23,6 |
| 30 | G1SXP5 | Homer scaffolding protein 1 | HOMER1 | 40,2 | 2,54 | 2 | 2 | 2 | Infinity |
| 31 | G1TNZ9 | Complexin 2 | CPLX2 | 14,98 | 8,33 | 1 | 1 | 1 | Infinity |
| 32 | G1T8W7 | V-type proton ATPase subunit a | ATP6V0A1 | 98,66 | 4,63 | 4 | 4 | 4 | Infinity |
| 33 | G1SKT4 | ATP synthase subunit alpha | ATP5F1A | 59,72 | 3,62 | 2 | 2 | 2 | Infinity |
| 34 | G1TCQ2 | Fatty acid binding protein 1 | FABP1 | 14,11 | 6,35 | 1 | 1 | 1 | Infinity |
| 35 | G1SE51 | Sperm associated antigen 9 | SPAG9 | 138,03 | 3,44 | 7 | 7 | 10 | Infinity |
| 36 | G1SZ00 | Cysteine and glycine rich protein 1 | CSRP1 | 20,58 | 13,99 | 2 | 2 | 2 | Infinity |
| 37 | G1SWY6 | Early endosome antigen 1 | EEA1 | 158,13 | 1,02 | 5 | 5 | 5 | 46,26 |
| 38 | G1U0T7 | SH3GL interacting endocytic adaptor 1 | SGIP1 | 26,365 | 5,02 | 3 | 3 | 3 | Infinity |
| 39 | G1SYI6 | Abl interactor 1 | ABI1 | 52,41 | 6,03 | 3 | 1 | 3 | Infinity |
| 40 | G1SUD8 | Abl interactor 2 | ABI2 | 55,45 | 8,59 | 4 | 2 | 4 | 37,9 |
| 41 | G1SHR3 | RUN and FYVE domain containing 1 | RUFY1 | 78,85 | 1,14 | 1 | 0 | 1 | 23,6 |
| 42 | G1SKP2 | Importin 5 | IPO5 | 120,56 | 1,03 | 2 | 2 | 3 | Infinity |
| 43 | G1TCR0 | Crystallin mu | CRYM | 33,59 | 3,18 | 1 | 1 | 1 | Infinity |
| 44 | G1TGQ6 | Phosphofurin acidic cluster sorting protein 1 | PACS1 | 98,62 | 1,56 | 2 | 2 | 3 | Infinity |
| 45 | G1T0H7 | Trafficking protein particle complex subunit | TRAPPC3 | 20,31 | 10,56 | 2 | 2 | 2 | Infinity |
| 46 | G1SL07 | Protein lin-7 homolog | LIN7C | 22 | 13,07 | 2 | 0 | 2 | Infinity |
| 47 | G1SY70 | Sec23 homolog A, coat complex II component | SEC23A | 86,148 | 3,92 | 6 | 4 | 6 | 89,07 |
| 48 | P06813 | Calpain small subunit 1 | CAPNS1 | 28,22 | 5,64 | 1 | 1 | 1 | Infinity |
| 49 | P06814 | Calpain-2 catalytic subunit (Fragment) | CAPN2 | 49,46 | 4,5 | 2 | 0 | 2 | Infinity |
| 50 | G1U6H0 | Calnexin | CANX | 67,71 | 3,88 | 3 | 3 | 3 | 51,5 |
| 51 | G1T2V6 | NSF attachment protein gamma | NAPG | 34,68 | 6,09 | 2 | 2 | 2 | 34,7 |
| 52 | G1SCP7 | IQ motif containing GTPase activating protein 1 | IQGAP1 | 189,19 | 1,09 | 5 | 5 | 5 | Infinity |
| 53 | G1SVI9 | VAMP associated protein A | VAPA | 32,54 | 9,56 | 2 | 2 | 2 | Infinity |
| 54 | G1SN27 | WASH complex subunit | WASHC4 | 134,39 | 0,69 | 2 | 2 | 2 | Infinity |
| Enzyme activity regulation | | | | | | | | | |
| 1 | G1SMP3 | Calcium/calmodulin dependent serine protein kinase | CASK | 104,4 | 1,2 | 4 | 4 | 4 | 34,21 |
| 2 | G1TDN4 | Protein kinase cAMP-dependent type I regulatory subunit alpha | PRKAR1A | 42,99 | 2,1 | 2 | 2 | 2 | 20,9 |
| 3 | G1SEJ8 | G protein subunit alpha i1 | GNAI1 | 40,32 | 3,11 | 1 | 1 | 1 | 27,79 |
| 4 | G1SK25 | Protein kinase cAMP-activated catalytic subunit beta | PRKACB | 42,19 | 2,45 | 2 | 2 | 4 | 31,52 |
| 5 | G1T512 | 2’,3’-cyclic nucleotide 3’ phosphodiesterase | CNP | 46,25 | 4,85 | 4 | 4 | 4 | 45,2 |
| 6 | G1SUS6 | Secretion associated Ras related GTPase 1B | SAR1B | 22,42 | 11,11 | 3 | 3 | 5 | Infinity |
| 7 | P10830 | Protein kinase C epsilon type | PRKCE | 83,46 | 2,58 | 3 | 2 | 3 | Infinity |
| 8 | Q6IVG4 | Arf-GAP with coiled-coil, ANK repeat and PH domain-containing protein 2 | ACAP2 | 87,85 | 1,41 | 3 | 3 | 4 | 31,5 |
| 9 | G1TUP1 | Programmed cell death 6 | PDCD6 | 22,16 | 5,76 | 3 | 3 | 4 | 26,2 |
| 10 | U3KNI3 | Reticulon | RTN1 | 23,47 | 21,63 | 8 | 3 | 12 | Infinity |
| 11 | G1SS91 | C4a anaphylatoxin | C4A | 192,68 | 1,14 | 4 | 4 | 4 | 49,7 |
| 12 | G1TUX8 | Non-specific serine/threonine protein kinase | PAK3 | 62,23 | 6,8 | 4 | 1 | 4 | Infinity |
| 13 | G1TEL6 | Calcium/calmodulin dependent protein kinase II alpha | CAMK2A | 59,91 | 13,91 | 8 | 6 | 8 | Infinity |
| 14 | G1U522 | Protein kinase cAMP-dependent type II regulatory subunit alpha | PRKAR2A | 45,03 | 7,73 | 4 | 3 | 4 | Infinity |
| 15 | G1TAD2 | Small ArfGAP2 | SMAP2 | 46,61 | 7,46 | 4 | 3 | 5 | Infinity |
| 16 | G1SWY0 | RAB6A, member RAS oncogene family | RAB6A | 23,5 | 14,42 | 3 | 2 | 7 | 46,92 |
| 17 | G1U7U8 | G protein subunit alpha o1 | GNAO1 | 34,59 | 6,95 | 2 | 2 | 2 | Infinity |
| 18 | G1SYI2 | G protein subunit beta 1 | GNB1 | 37,35 | 12,65 | 4 | 1 | 4 | 23,5 |
| 19 | G1T5L2 | Retinaldehyde binding protein 1 | RLBP1 | 36,4 | 0 | 3 | 3 | 3 | Infinity |
| 20 | G1SGT7 | MAP kinase activating death domain | MADD | 182,91 | 0,49 | 2 | 2 | 2 | 24,9 |
| 21 | G1T9N9 | Mitogen-activated protein kinase | MAPK9 | 44,46 | 2,05 | 1 | 1 | 2 | 20,8 |
| 22 | G1T5Z7 | Mitogen-activated protein kinase | MAPK3 | 40,27 | 13,75 | 7 | 1 | 8 | Infinity |
| 23 | G1SCX2 | DIRAS family GTPase 2 | DIRAS2 | 22,46 | 10,05 | 3 | 3 | 3 | Infinity |
| 24 | G1U3T2 | MAP7 domain containing 1 | MAP7D1 | 89,32 | 1,48 | 1 | 1 | 2 | 29,7 |
| 25 | G1T140 | Protein kinase C and casein kinase substrate in neurons 1 | PACSIN1 | 50,67 | 10,59 | 5 | 5 | 5 | Infinity |
| 26 | G1TS93 | Protein phosphatase 1 regulatory subunit 7 | PPP1R7 | 39,95 | 2,6 | 2 | 2 | 2 | 25,6 |
| 27 | G1SM05 | Secretion associated Ras related GTPase 1A | SAR1A | 22,38 | 6,06 | 3 | 1 | 4 | 29,7 |
| 28 | G1SXL3 | TPR_REGION domain-containing protein | ST13 | 41,28 | 12,47 | 5 | 5 | 5 | Infinity |
| 29 | Q28653 | Serine/threonine-protein phosphatase 2A 56 kDa regulatory subunit delta isoform | PPP2R5D | 68,05 | 7,85 | 5 | 5 | 6 | Infinity |
| 30 | G1T2M0 | KRAS proto-oncogene, GTPase | KRAS | 26,71 | 4,53 | 1 | 1 | 1 | Infinity |
| 31 | G1SZ94 | ArfGAP with FG repeats 1 | AGFG1 | 51,94 | 2,17 | 1 | 1 | 1 | Infinity |
| 32 | G1T6P5 | ADP ribosylation factor like GTPase 8B | ARL8B | 16,73 | 6,21 | 1 | 1 | 1 | Infinity |
| 33 | G1T2I5 | Reticulon | RTN4 | 112,65 | 0,97 | 3 | 3 | 4 | 24 |
| 34 | G1SIG2 | CRK proto-oncogene, adaptor protein | CRK | 22,93 | 4,9 | 2 | 1 | 2 | Infinity |
| Protective proteins | | | | | | | | | |
| 1 | P01847 | Ig lambda chain C region | N/A | 11,48 | 16,19 | 1 | 1 | 1 | Infinity |
| 2 | G1T6I6 | Interleukin enhancer binding factor 3 | ILF3 | 95,82 | 7,34 | 6 | 6 | 7 | Infinity |
| 3 | G1T310 | Family with sequence similarity 213 member A | FAM213A | 25,49 | 4,8 | 3 | 3 | 3 | 30,8 |
| 4 | G1SDC4 | Oxidation resistance 1 | OXR1 | 96,64 | 4,98 | 7 | 7 | 7 | Infinity |
| 5 | G1TZE2 | Glutathione S-transferase zeta 1 | GSTZ1 | 24,26 | 11,06 | 1 | 1 | 1 | Infinity |
| 6 | G1SST9 | UV excision repair protein RAD23 | RAD23B | 43,25 | 7,33 | 5 | 5 | 6 | Infinity |
| 7 | G1U1F6 | Heat shock protein family B (small) member 6 | HSPB6 | 17,31 | 20,99 | 3 | 3 | 4 | Infinity |
| Protein regulators of gene expression, cell division, and differentiation | | | | | | | | | |
| 1 | G1SUU7 | tRNA-splicing ligase RtcB homolog | RTCB | 53,77 | 7,35 | 3 | 3 | 3 | 49 |
| 2 | G1SF95 | Heterogeneous nuclear ribonucleoprotein D | HNRNPD | 31,54 | 8,66 | 2 | 1 | 2 | Infinity |
| 3 | G1T8P3 | Small nuclear ribonucleoprotein U1 subunit 70 | SNRNP70 | 51,44 | 2,41 | 3 | 3 | 3 | 26,3 |
| 4 | G1TE76 | Eukaryotic translation initiation factor 4H | EIF4H | 27,37 | 5,26 | 1 | 1 | 1 | Infinity |
| 5 | G1SS73 | DEAD-box helicase 1 | DDX1 | 82,39 | 1,08 | 1 | 1 | 1 | 23,7 |
| 6 | G1TA11 | Arginyl-tRNA synthetase | RARS | 75,6 | 1,82 | 1 | 1 | 2 | 29,7 |
| 7 | G1SY68 | Splicing factor 3b subunit 2 | SF3B2 | 95,81 | 1,41 | 1 | 1 | 1 | Infinity |
| 8 | G1SL60 | Splicing factor 3b subunit 3 | SF3B3 | 135,12 | 2,14 | 3 | 3 | 4 | 49,9 |
| 9 | G1TKC4 | Lysyl-tRNA synthetase | KARS | 61,98 | 4,04 | 2 | 2 | 2 | 34 |
| 10 | G1SFV7 | Damage specific DNA binding protein 1 | DDB1 | 126,83 | 1,75 | 4 | 4 | 4 | Infinity |
| 11 | G1T0B0 | Chromosome 11 open reading frame 68 | C11orf68 | 31,16 | 3,46 | 2 | 2 | 2 | Infinity |
| 12 | G1SMZ5 | Eukaryotic translation initiation factor 3 subunit A | EIF3A | 164,475 | 1,84 | 5 | 5 | 5 | 42,1 |
| 13 | G1SPW3 | ELAV-like protein | ELAVL2 | 42,52 | 8,51 | 4 | 0 | 4 | 48,5 |
| 14 | G1TI40 | Small nuclear ribonucleoprotein D2 polypeptide | SNRPD2 | 13,52 | 8,47 | 2 | 2 | 2 | 34,2 |
| 15 | G1SVP9 | Poly(A) binding protein nuclear 1 | PABPN1 | 32,12 | 3,67 | 1 | 1 | 1 | 24 |
| 16 | G1TGK9 | DExH-box helicase 9 | DHX9 | 139,39 | 0,95 | 2 | 2 | 3 | Infinity |
| 17 | U3KNL5 | Eukaryotic translation initiation factor 3 subunit F | EIF3F | 37,79 | 3,02 | 2 | 2 | 2 | Infinity |
| 18 | G1TBS2 | RuvB-like helicase | RUVBL1 | 50,2 | 2,19 | 2 | 2 | 3 | Infinity |
| 19 | G1SDN4 | Synaptotagmin binding cytoplasmic RNA interacting protein | SYNCRIP | 62,62 | 3,56 | 2 | 0 | 2 | 28,2 |
| 20 | G1SSX5 | Methionyl-tRNA synthetase | MARS | 101,1 | 2,56 | 3 | 3 | 3 | Infinity |
| 21 | G1SHL8 | Signal transducer and activator of transcription | STAT1 | 88,12 | 7,8 | 6 | 6 | 7 | Infinity |
| 22 | G1T8S4 | Clustered mitochondria protein homolog | CLUH | 147,95 | 0,83 | 2 | 2 | 2 | 26,72 |
| 23 | G1TE26 | NOVA alternative splicing regulator 1 | NOVA1 | 51,71 | 2,76 | 1 | 0 | 1 | 21 |
| 24 | G1U1M3 | Aminoacyl tRNA synthetase complex interacting multifunctional protein 1 | AIMP1 | 35,23 | 2,51 | 3 | 3 | 3 | 27,9 |
| 25 | G1TD91 | Cleavage and polyadenylation specificity factor subunit 5 | NUDT21 | 26,22 | 7,93 | 1 | 1 | 1 | Infinity |
| 26 | G1T586 | Helix-destabilizing protein | HNRNPA1 | 32,81 | 10,32 | 3 | 0 | 6 | 26,77 |
| 27 | G1SKE2 | TIA1 cytotoxic granule associated RNA binding protein like 1 | TIAL1 | 41,37 | 2,94 | 1 | 1 | 1 | 27,72 |
| 28 | G1U636 | Cell cycle associated protein 1 | CAPRIN1 | 78,36 | 1,55 | 1 | 1 | 1 | Infinity |
| 29 | G1SIP2 | Heterogeneous nuclear ribonucleoprotein L | HNRNPL | 64,03 | 5,29 | 3 | 3 | 3 | Infinity |
| 30 | G1SM70 | Enhancer of rudimentary homolog |  | 12,25 | 21,15 | 2 | 2 | 2 | Infinity |
| 31 | G1SE50 | CRK like proto-oncogene, adaptor protein | CRKL | 33,82 | 8,25 | 3 | 2 | 3 | Infinity |
| 32 | G1TCK9 | Isoleucyl-tRNA synthetase | IARS | 144,77 | 1,58 | 3 | 3 | 3 | 38,6 |
| 33 | G1T0N5 | Purine rich element binding protein A | PURA | 32,84 | 9,15 | 3 | 3 | 3 | 68,1 |

**Table 4. Proteins of 20S proteasome fraction (Rabbit brain) identified in two independent experiments**

| # | Main Accession | Description | Gene Name | MW [kDa] | Confident Coverage [%] | #Peptides | #Unique | #PSMs | Score | |
| --- | --- | --- | --- | --- | --- | --- | --- | --- | --- | --- |
| Core particle subunits | | | | | | | | | |  |
| 1 | G1SVF2 | 26S proteasome non-ATPase regulatory subunit 1 | PSMD1 | 108,43 | 0 | 1 | 1 | 3 | Infinity | |
| 2 | G1SQL0 | AAA domain-containing protein | N/A | 49,15 | 2,73 | 1 | 1 | 1 | Infinity | |
| Other components of UPS | | | | | | | | | |  |
| 1 | G1SS07 | UBC core domain-containing protein | UBE2V2 | 16,69 | 12,93 | 2 | 1 | 4 | Infinity | |
| 2 | G1SW41 | Ubiquitin conjugating enzyme E2 D4 (putative) | UBE2D4 | 16,84 | 7,38 | 1 | 1 | 1 | 32,92 | |
| 3 | G1TTU6 | S-phase kinase-associated protein 1 | N/A | 18,65 | 7,36 | 1 | 1 | 1 | Infinity | |
| 4 | G1SYL8 | Elongin B | ELOB | 12,54 | 15,04 | 3 | 3 | 4 | Infinity | |
| Metabolic enzymes | | | | | | | | | |  |
| 1 | G1T7V5 | Dihydrolipoyl dehydrogenase | DLD | 54,3 | 2,56 | 1 | 1 | 2 | Infinity | |
| 2 | G1T013 | Malic enzyme | ME1 | 64,11 | 1,92 | 2 | 2 | 2 | 38,09 | |
| 3 | G1SQP9 | Catechol-O-methyltransferase | COMT | 29,87 | 8,21 | 3 | 3 | 3 | Infinity | |
| 4 | G1T6W7 | Catalase | CAT | 59,62 | 4,55 | 3 | 3 | 3 | Infinity | |
| 5 | G1SK42 | Transglutaminase 2 | TGM2 | 82,49 | 0 | 4 | 4 | 4 | 20,77 | |
| 6 | G1SPZ9 | Inorganic diphosphatase | PPA2 | 38,7 | 3,81 | 1 | 1 | 1 | Infinity | |
| 7 | G1SEV2 | Protein disulfide-isomerase | PDIA3 | 75,04 | 19,71 | 12 | 12 | 15 | Infinity | |
| 8 | G1TCA8 | Carbonyl reductase 3 | CBR3 | 30,68 | 8,3 | 2 | 0 | 3 | Infinity | |
| 9 | G1TUA3 | Peptidase A1 domain-containing protein | N/A | 44,83 | 4,32 | 3 | 3 | 5 | Infinity | |
| 10 | G1U6H4 | Dimethylarginine dimethylaminohydrolase 2 | DDAH2 | 29,85 | 11,11 | 3 | 2 | 3 | 46,1 | |
| 11 | G1SQS1 | Aldose 1-epimerase | GALM | 37,59 | 4,09 | 1 | 1 | 1 | 20,56 | |
| 12 | G1T7L5 | Inositol-1-monophosphatase | IMPA1 | 31,05 | 7,72 | 3 | 3 | 3 | Infinity | |
| 13 | G1SE67 | Sepiapterin reductase | SPR | 28,25 | 19,01 | 4 | 4 | 4 | Infinity | |
| 14 | P82810 | Morphine 6-dehydrogenase (Fragments) | N/A | 14,1 | 7,2 | 3 | 0 | 4 | Infinity | |
| 15 | G1TDQ5 | ATP-dependent 6-phosphofructokinase | PFKM | 85,15 | 22,82 | 23 | 0 | 35 | 32,25 | |
| 16 | G1T2V2 | AIR carboxylase | PAICS | 47,44 | 5,14 | 3 | 3 | 3 | 55,23 | |
| 17 | G1T465 | 3alpha(17beta)-hydroxysteroid dehydrogenase | PGER6 | 36,63 | 2,79 | 2 | 0 | 4 | Infinity | |
| 18 | G1TGF1 | Prostaglandin E synthase 3 | PTGES3 | 18,69 | 6,25 | 1 | 1 | 3 | Infinity | |
| 19 | G1SQA1 | Carboxymethylenebutenolidase homolog | CMBL | 27,93 | 24,9 | 6 | 6 | 10 | Infinity | |
| 20 | G1TLA5 | 3-oxo-5-beta-steroid 4-dehydrogenase | AKR1D1 | 37,14 | 9,2 | 5 | 0 | 7 | Infinity | |
| 21 | G1SZ37 | 3-hydroxyisobutyrate dehydrogenase | HIBADH | 35,31 | 3,87 | 1 | 1 | 1 | Infinity | |
| 22 | G1SIF2 | Phosphoenolpyruvate carboxykinase 2, mitochondrial | PCK2 | 70,57 | 9,53 | 6 | 6 | 6 | Infinity | |
| 23 | G1TPZ3 | Fumarylacetoacetate hydrolase domain containing 2A | FAHD2A | 34,34 | 7,96 | 2 | 2 | 2 | 45,3 | |
| 24 | G1TQI2 | Dihydropyrimidinase like 4 | DPYSL4 | 61,22 | 2,8 | 2 | 2 | 2 | Infinity | |
| 25 | G1U5N5 | Lactamase_B domain-containing protein | N/A | 18,6 | 26,29 | 3 | 3 | 3 | Infinity | |
| 26 | G1U3M5 | Protein arginine methyltransferase 1 | PRMT1 | 42,35 | 5,66 | 2 | 2 | 3 | Infinity | |
| 27 | Q9TTJ6 | Regucalcin | RGN | 33,09 | 12,04 | 5 | 5 | 5 | Infinity | |
| 28 | G1T512 | 2',3'-cyclic nucleotide 3' phosphodiesterase | CNP | 46,25 | 7,77 | 4 | 4 | 5 | Infinity | |
| 29 | G1TA95 | Phosphoribosyl pyrophosphate synthetase associated protein 2 | PRPSAP2 | 39,54 | 3,91 | 1 | 0 | 1 | Infinity | |
| 30 | G1TCW5 | Peptidase D | PEPD | 54,63 | 1,83 | 2 | 2 | 2 | Infinity | |
| 31 | G1SYS6 | Acylphosphatase | ACYP1 | 16,55 | 8,39 | 1 | 1 | 1 | 33,17 | |
| 32 | G1TXN1 | Nitrilase family member 2 | NIT2 | 31,3 | 5,3 | 2 | 2 | 2 | Infinity | |
| 33 | G1T726 | Hydroxyacyl-CoA dehydrogenase | HADH | 34,34 | 5,41 | 4 | 4 | 4 | Infinity | |
| 34 | G1TB17 | Adenylosuccinate lyase | ADSL | 47,44 | 2,62 | 1 | 1 | 1 | 19,98 | |
| 35 | G1TES6 | Hydroxysteroid 17-beta dehydrogenase 10 | HSD17B10 | 27,08 | 26,82 | 6 | 6 | 7 | Infinity | |
| 36 | G1SP48 | UDP-glucose 6-dehydrogenase | UGDH | 53,62 | 4,15 | 3 | 3 | 3 | Infinity | |
| 37 | P07952 | Bisphosphoglycerate mutase | BPGM | 30,01 | 4,63 | 1 | 1 | 1 | 20,6 | |
| 38 | Q95KM0 | Arginase-1 | ARG1 | 34,43 | 7,45 | 3 | 3 | 3 | Infinity | |
| 39 | G1TK30 | Cysteine sulfinic acid decarboxylase | CSAD | 56,11 | 2,39 | 1 | 1 | 1 | Infinity | |
| 40 | G1T616 | Phosphoglucomutase 2 | PGM2 | 68,66 | 1,78 | 2 | 2 | 3 | Infinity | |
| 41 | G1SKT4 | ATP synthase subunit alpha | ATP5F1A | 59,72 | 2,17 | 3 | 3 | 3 | 52,28 | |
| 42 | G1SH60 | Acyl-CoA synthetase short chain family member 2 | ACSS2 | 79,02 | 1,28 | 1 | 1 | 1 | 20,6 | |
| 43 | G1T7S1 | Aldo_ket_red domain-containing protein | N/A | 37,25 | 2,41 | 1 | 1 | 1 | 25,38 | |
| 44 | G1SCQ1 | Aldo-keto reductase family 7 like (gene/pseudogene) | AKR7L | 39,84 | 6,59 | 2 | 2 | 2 | 33,05 | |
| 45 | G1T218 | 4-hydroxyphenylpyruvate dioxygenase | HPD | 44,8 | 4,33 | 5 | 5 | 5 | 60,81 | |
| 46 | Q9GKX2 | Dehydrogenase/reductase SDR family member 4 (Fragment) | DHRS4 | 27,41 | 5 | 1 | 0 | 1 | Infinity | |
| 47 | G1SYE0 | Abhydrolase domain containing 14B | ABHD14B | 22,41 | 20,48 | 5 | 5 | 6 | Infinity | |
| 48 | G1TBU9 | Acetyl-CoA acyltransferase 2 | ACAA2 | 41,97 | 14,61 | 5 | 5 | 5 | Infinity | |
| 49 | O46640 | Amine sulfotransferase | SULT3A1 | 35,92 | 6,98 | 2 | 0 | 3 | 58,07 | |
| 50 | G1SL42 | Nipsnap homolog 1 | NIPSNAP1 | 33,32 | 3,17 | 1 | 1 | 1 | 22,3 | |
| 51 | G1SPY4 | Asparaginase like 1 | ASRGL1 | 33,78 | 8,02 | 2 | 2 | 2 | Infinity | |
| Components of cytoskeleton | | | | | | | | | |  |
| 1 | P63169 | Dynein light chain 1, cytoplasmic | DYNLL1 | 10,36 | 12,36 | 1 | 0 | 2 | Infinity | |
| 2 | G1SVF4 | Dynein light chain roadblock | DYNLRB2 | 10,84 | 9,38 | 1 | 1 | 1 | Infinity | |
| 3 | G1TF67 | Coronin | CORO1A | 49,36 | 17,04 | 10 | 10 | 12 | Infinity | |
| 4 | G1U949 | Microtubule associated protein RP/EB family member 1 | MAPRE1 | 30 | 10,07 | 2 | 1 | 2 | 22,35 | |
| 5 | G1TAM3 | Tubulin folding cofactor | TBCB | 27,4 | 12,3 | 3 | 3 | 3 | Infinity | |
| 6 | G1SMX2 | Internexin neuronal intermediate filament protein alpha | INA | 55,17 | 2,41 | 2 | 2 | 2 | Infinity | |
| Proteins of signal transduction and trafficking | | | | | | | | | |  |
| 1 | G1T8C8 | Sorting nexin 12 | SNX12 | 19,16 | 12,05 | 5 | 4 | 6 | 77,94 | |
| 2 | G1SFC1 | Golgin A4 | GOLGA4 | 249,81 | 0 | 8 | 8 | 9 | 32,28 | |
| 3 | G1SZ91 | Fatty acid binding protein 5 | FABP5 | 12,65 | 15,93 | 2 | 2 | 3 | 52,49 | |
| 4 | G1U2E3 | Charged multivesicular body protein 4B | CHMP4B | 24,96 | 4,46 | 1 | 1 | 1 | Infinity | |
| 5 | O97862 | Cystatin-C | CST3 | 16,34 | 7,43 | 1 | 1 | 1 | Infinity | |
| 6 | G1SJ77 | Chloride intracellular channel protein | CLIC4 | 24,09 | 5,69 | 1 | 1 | 1 | 23,62 | |
| 7 | G1T2C4 | Transgelin | TAGLN | 23,93 | 14,42 | 3 | 3 | 3 | Infinity | |
| 8 | G1SDA2 | Cellular retinoic acid binding protein 1 | CRABP1 | 15,58 | 10,22 | 1 | 1 | 1 | Infinity | |
| 9 | G1SDB9 | SEC14 like lipid binding 2 | SEC14L2 | 44,2 | 5,21 | 2 | 2 | 2 | 33,24 | |
| 10 | G1T4G9 | Tropomodulin 2 | TMOD2 | 39,43 | 2,56 | 3 | 3 | 3 | 25,45 | |
| 11 | G1SG31 | Nipsnap homolog 2 | NIPSNAP2 | 32,63 | 3,26 | 2 | 2 | 2 | 30,5 | |
| 12 | G1SL62 | Annexin | ANXA2 | 39,24 | 13,01 | 8 | 8 | 8 | Infinity | |
| 13 | G1TA83 | Annexin | ANXA4 | 35,83 | 7,84 | 2 | 2 | 2 | Infinity | |
| 14 | P07489 | Transthyretin | TTR | 13,65 | 9,45 | 1 | 1 | 2 | Infinity | |
| 15 | G1TR45 | EF-hand domain family member D2 | EFHD2 | 17,32 | 7,59 | 1 | 1 | 1 | Infinity | |
| 16 | G1TBZ1 | GABA type A receptor associated protein like 2 | GABARAPL2 | 13,66 | 9,4 | 2 | 2 | 2 | Infinity | |
| 17 | G1TKR2 | Synapsin I | SYN1 | 73,23 | 7,22 | 4 | 4 | 4 | Infinity | |
| 18 | G1TSU5 | Purine rich element binding protein B | PURB | 31,18 | 6,01 | 2 | 0 | 2 | 52,54 | |
| 19 | G1TH33 | Electron transfer flavoprotein beta subunit | ETFB | 25,74 | 17,3 | 4 | 4 | 4 | 106,4 | |
| 20 | G1U3U2 | Calsyntenin 1 | CLSTN1 | 114,65 | 2,12 | 4 | 4 | 4 | Infinity | |
| 21 | G1SFE4 | Synaptojanin-1 | SYNJ1 | 170,67 | 1,42 | 10 | 10 | 13 | Infinity | |
| 22 | G1U8W5 | Brevican | BCAN | 95,29 | 0 | 1 | 1 | 1 | 33,01 | |
| 23 | G1TJW8 | Pyridoxal phosphate homeostasis protein | PLPBP | 28,26 | 3,53 | 2 | 2 | 2 | 26,51 | |
| Enzyme activity regulation | | | | | | | | | |  |
| 1 | G1T6B3 | Serine/threonine kinase receptor associated protein | STRAP | 38,48 | 7,43 | 2 | 2 | 2 | Infinity | |
| 2 | G1T2M0 | KRAS proto-oncogene, GTPase | KRAS | 26,71 | 4,53 | 1 | 1 | 1 | Infinity | |
| 3 | G1SF47 | Septin 11 | SEPT11 | 53,99 | 4,88 | 3 | 3 | 5 | 32,11 | |
| 4 | G1SFU0 | Ras-related protein Rab-7a | RAB7A | 23,5 | 21,26 | 5 | 5 | 7 | Infinity | |
| 5 | O97572 | Ras-related protein Rab-7a | RAB7A | 23,53 | 15,46 | 4 | 4 | 4 | 91,16 | |
| 6 | P62493 | Ras-related protein Rab-11A | RAB11A | 24,38 | 10,19 | 2 | 2 | 2 | Infinity | |
| 7 | G1SS79 | Myristoylated alanine rich protein kinase C substrate | MARCKS | 31,73 | 5,41 | 2 | 2 | 2 | Infinity | |
| 8 | G1SK25 | Protein kinase cAMP-activated catalytic subunit beta | PRKACB | 42,19 | 7,63 | 5 | 5 | 6 | Infinity | |
| 9 | U3KPB1 | Protein phosphatase, Mg2+/Mn2+ dependent 1H | PPM1H | 56,31 | 2,91 | 2 | 2 | 2 | Infinity | |
| 10 | G1SYC9 | RAB8B, member RAS oncogene family | RAB8B | 29,81 | 12,69 | 4 | 2 | 7 | Infinity | |
| 11 | G1SGI5 | RAB3B, member RAS oncogene family | RAB3B | 24,64 | 10,05 | 4 | 3 | 6 | Infinity | |
| 12 | U3KMP1 | RAB10, member RAS oncogene family | RAB10 | 25,91 | 14,72 | 3 | 1 | 6 | 32,49 | |
| 13 | G1TD28 | Small ArfGAP 1 | SMAP1 | 50,67 | 2,35 | 1 | 1 | 1 | Infinity | |
| 14 | G1T1N2 | Calcium/calmodulin dependent protein kinase ID | CAMK1D | 40,52 | 3,01 | 2 | 2 | 2 | Infinity | |
| Protective proteins | | | | | | | | | |  |
| 1 | G1T4A7 | Glutathione transferase | N/A | 29,29 | 19,07 | 9 | 4 | 12 | Infinity | |
| 2 | G1TDH4 | Thioredoxin domain-containing protein | N/A | 27,48 | 5,51 | 1 | 1 | 1 | 33,19 | |
| 3 | G1T4P8 | Glutaredoxin 3 | GLRX3 | 32,36 | 7,32 | 3 | 3 | 3 | 45,47 | |
| 4 | G1SRN2 | NmrA domain-containing protein | N/A | 34,37 | 8,06 | 4 | 4 | 4 | Infinity | |
| Protein regulators of gene expression, cell division, and differentiation | | | | | | | | | |  |
| 1 | G1SYB4 | Cell division control protein 42 homolog | CDC42 | 22,83 | 9,27 | 2 | 2 | 2 | Infinity | |
| 2 | G1SMM9 | Growth associated protein 43 | GAP43 | 24,27 | 17,45 | 3 | 3 | 3 | Infinity | |
| 3 | G1T0F0 | Growth arrest specific 7 | GAS7 | 47,62 | 4,81 | 2 | 2 | 2 | 43,45 | |
| 4 | G1T2G4 | Eukaryotic translation initiation factor 2 subunit 1 | EIF2S1 | 36,09 | 6,35 | 3 | 3 | 3 | Infinity | |
| 5 | P02252 | Histone H1.4 | HIST1H1E | 21,88 | 5,02 | 1 | 1 | 1 | Infinity | |
| 6 | G1SI29 | Elongation factor Tu | TUFM | 49,65 | 4,61 | 4 | 4 | 4 | Infinity | |
| 7 | P29562 | Eukaryotic initiation factor 4A-I (Fragment) | EIF4A1 | 45,26 | 20,6 | 11 | 3 | 14 | Infinity | |
| 8 | P47814 | Eukaryotic translation initiation factor 1A | EIF1A | 16,52 | 7,64 | 1 | 1 | 1 | 19,98 | |
| 9 | G1TE69 | Serine/arginine-rich splicing factor 1 | SRSF1 | 52,04 | 2,09 | 1 | 1 | 1 | 33,17 | |
| 10 | G1T3Y3 | Chromosome 20 open reading frame 27 | C20orf27 | 27,88 | 3,89 | 1 | 1 | 1 | Infinity | |
| 11 | P53787 | Elongation factor 1-delta | EEF1D | 31,06 | 12,5 | 4 | 4 | 4 | Infinity | |
| 12 | G1SZJ5 | Prefoldin subunit 6 | PFDN6 | 14,57 | 9,3 | 3 | 3 | 3 | 35,03 | |
| 13 | G1U7Q6 | Translin | TSN | 26,15 | 4,39 | 1 | 1 | 1 | Infinity | |
